# Genome-wide screen identifies host loci that modulate *Mycobacterium tuberculosis* fitness in immunodivergent mice

**DOI:** 10.1093/g3journal/jkad147

**Published:** 2023-07-05

**Authors:** Rachel K Meade, Jarukit E Long, Adrian Jinich, Kyu Y Rhee, David G Ashbrook, Robert W Williams, Christopher M Sassetti, Clare M Smith

**Affiliations:** Department of Molecular Genetics and Microbiology, Duke University, Durham, NC 27710, USA; University Program in Genetics and Genomics, Duke University, Durham, NC 27710, USA; Department of Microbiology and Physiological Systems, UMass Chan Medical School, Worcester, MA 01655, USA; Research Animal Diagnostic Services, Charles River Laboratories, Wilmington, MA 01887, USA; Division of Infectious Diseases, Weill Cornell Medical College, New York, NY 10021, USA; Division of Infectious Diseases, Weill Cornell Medical College, New York, NY 10021, USA; Department of Genetics, Genomics and Informatics, University of Tennessee Health Science Center, Memphis, TN 38163, USA; Department of Genetics, Genomics and Informatics, University of Tennessee Health Science Center, Memphis, TN 38163, USA; Department of Microbiology and Physiological Systems, UMass Chan Medical School, Worcester, MA 01655, USA; Department of Molecular Genetics and Microbiology, Duke University, Durham, NC 27710, USA; University Program in Genetics and Genomics, Duke University, Durham, NC 27710, USA

**Keywords:** host–pathogen interactions, tuberculosis, mycobacteria, systems genetics, genomics, genetic diversity, natural variation, bacterial genetics, TnSeq, mouse models, BXD, QTL mapping, complex traits

## Abstract

Genetic differences among mammalian hosts and among strains of *Mycobacterium tuberculosis* (*Mtb*) are well-established determinants of tuberculosis (TB) patient outcomes. The advent of recombinant inbred mouse panels and next-generation transposon mutagenesis and sequencing approaches has enabled dissection of complex host–pathogen interactions. To identify host and pathogen genetic determinants of *Mtb* pathogenesis, we infected members of the highly diverse BXD family of strains with a comprehensive library of *Mtb* transposon mutants (TnSeq). Members of the BXD family segregate for *Mtb*-resistant C57BL/6J (B6 or *B*) and *Mtb*-susceptible DBA/2J (D2 or *D*) haplotypes. The survival of each bacterial mutant was quantified within each BXD host, and we identified those bacterial genes that were differentially required for *Mtb* fitness across BXD genotypes. Mutants that varied in survival among the host family of strains were leveraged as reporters of “endophenotypes,” each bacterial fitness profile directly probing specific components of the infection microenvironment. We conducted quantitative trait loci (QTL) mapping of these bacterial fitness endophenotypes and identified 140 *host–pathogen* QTL (*hp*QTL). We located a QTL hotspot on chromosome 6 (75.97–88.58 Mb) associated with the genetic requirement of multiple *Mtb* genes: *Rv0127* (*mak*), *Rv0359* (*rip2*), *Rv0955* (*perM*), and *Rv3849* (*espR*). Together, this screen reinforces the utility of bacterial mutant libraries as precise reporters of the host immunological microenvironment during infection and highlights specific host–pathogen genetic interactions for further investigation. To enable downstream follow-up for both bacterial and mammalian genetic research communities, all bacterial fitness profiles have been deposited into GeneNetwork.org and added into the comprehensive collection of TnSeq libraries in MtbTnDB.

## Introduction

Every pathogenic infection is part of an evolutionary battle between host and invader. In the case of *Mycobacterium tuberculosis* (*Mtb*), the causative agent of tuberculosis (TB), evidence tracing back at least 9,000 years tells the tale of a host–pathogen arms race (Hershkovitz *et al*. 2008), making *Mtb* one of the most enduring adversaries of the human species. Given the prevailing nature of this challenge, it is no surprise that weaknesses in vital host defenses provide greater opportunity for *Mtb* infection, allow *Mtb* to manipulate the nature and magnitude of the host immune response, and worsen patient prognoses (Cooper *et al*. 1993; Flynn *et al*. 1993; Cooper *et al*. 1997; Caruso *et al*. 1999; Kramnik *et al*. 2000). In addition, the advent of drug-resistant and multidrug-resistant *Mtb* strains highlights the imminent danger posed by our microscopic rivals and underscores the importance of bacterial variation as a critical factor in the pathogenesis and continued spread of *Mtb* (Caws *et al*. 2008; Hernández-Pando *et al*. 2012; Gopal *et al*. 2014). Meanwhile, genetic diversity of both hosts and *Mtb* strains that interact during infection can give rise to a spectrum of disease outcomes (Smith and Sassetti 2018), enhancing the complexity of TB identification and treatment. To combat a pathogen responsible for nearly 10.6 million infections and 1.6 million deaths annually (WHO 2022), it is therefore vital to dissect the host–pathogen interface.

Mammalian models of TB have been used successfully to identify and mechanistically interrogate TB susceptibility loci identified from human cohorts. For mouse strains that are phenotypically divergent, quantitative trait locus (QTL) mapping studies within genetically tractable and reproducible hosts have enabled the identification of genomic loci that contribute to the magnitude of clinically relevant phenotypes (Lavebratt *et al*. 1999; Kramnik *et al*. 2000; Mitsos *et al*. 2000; Mitsos *et al*. 2003; Yan *et al*. 2006). The classic inbred strains C57BL/6J (B6) and DBA/2J (D2) lie on opposite ends of the TB susceptibility spectrum, with B6 surviving nearly a year postinfection and D2 succumbing to cachexia and morbidity within months (Medina and North 1998). To capitalize on this diverse disease outcome and just over 6 million divergent SNPs (Wang *et al*. 2016; Ashbrook *et al*. 2021; Ashbrook *et al*. 2022; Sasani *et al*. 2022), a recombinant inbred panel generated from these 2 founders, known as the BXD, has laid the foundation for understanding host determinants of susceptibility to a variety of diseases through QTL mapping in genetically diverse mice (Taylor *et al*. 1973; Taylor *et al*. 1999; Peirce *et al*. 2004; Ashbrook *et al*. 2021).

BXD and other recombinant inbred panels have been used to explore the intricate dynamics of TB disease, but often the phenotypes leveraged to understand these dynamics in mice, such as survival time postinfection, body weight, or bacterial burden, are too complex and polygenic to reveal novel biology or enable refined mapping. These “macrophenotypes” materialize from many genetic and environmental factors that can be difficult to mechanistically dissect or precisely map. Within each unique host, bacteria face similarly diverse immunological dynamics and spatiotemporal selective pressures. To contribute to the growing body of work on genetic determinants of TB outcome, quantifying precise bacterial “endophenotypes” underlying disease outcomes is the crucial next step toward elaborating the biological intricacies that govern the host-pathogen interface. Recently employed molecular endophenotypes include expression QTL (Chesler *et al*. 2005), metabolite QTL (Wu *et al*. 2014), protein QTL (Chick *et al*. 2016), and chromatin accessibility QTL (Skelly *et al*. 2020), but for infectious disease, the most salient endophenotypes lie at the interface of the host and pathogen.

A classic approach to assess the requirement of a bacterial gene for infection is to infect a standard inbred mouse strain, such as B6, with a single knockout *Mtb* mutant. Expanding on this design, genome-wide mutagenesis enables the generation of single knockout mutants across the *Mtb* genome, thereby allowing the measurement of *Mtb* gene requirements under a variety of selective pressures. Transposon (Tn) mutant libraries (TnSeq) have been successfully used to report on variable components of the bacterial infection microenvironment under distinct host and antibiotic pressures (Sassetti and Rubin 2003; Nambi *et al*. 2015; Mishra *et al*. 2017; Bellerose *et al*. 2019). We have recently leveraged TnSeq across genetically diverse Collaborative Cross (CC) mice to map both host- and pathogen-linked loci (Smith *et al*. 2022). Deep phenotyping of disease traits in over 50 CC strains enabled mapping of 10 *tuberculosis immunophenotype* QTL (*Tip*QTL), derived from bacterial burden and cytokine quantifications in each host. Measurement of TnSeq mutant fitness during in vivo infection yielded 46 *host interacting with pathogen* QTL (*Hip*QTL). While 8 founder lines do provide a greater extent of SNP-level diversity in the CC panel than in the BXD panel (Munger *et al*. 2014), the additional founders can reduce statistical strength for QTL mapping and complicate determination of allele effects for mapped QTL. The 2-state model of the BXD panel allows relatively straightforward identification of significant QTL and downstream allele effects, potentially shortening the path from screen to biological discovery.

To further characterize the BXD phenome (Chesler *et al*. 2004) and add to the growing repertoire of selective pressures applied to *Mtb* whole-genome mutant libraries (Jinich *et al*. 2021), we now leverage the phenotypic divergence and reproducibility of the BXD family and the molecular phenotyping precision of an *Mtb* Tn mutant library. We infected 19 BXD strains and both parents with a saturated library of *Mtb* Tn mutants and conducted dual-genome QTL mapping to identify linkages between bacterial fitness profiles and host genetic factors, revealing 140 host genetic loci significantly associated with specific bacterial mutants. Thus, in passing a bacterial Tn mutant library through selection within the immunologically and phenotypically diverse BXD microenvironments, we have conducted a multidimensional host–pathogen screen to identify precise host and bacterial determinants of TB disease. Overall, this study provides a blueprint for the dissection of host–pathogen interactions, which can play a pivotal role in functional annotation of understudied host and bacterial genes.

## Materials and methods

### Ethics statement

All mouse studies were conducted in accordance with the guidelines issued in the Guide for the Care and Use of Laboratory Animals of the National Institutes of Health and the Office of Laboratory Animal Welfare. Animal studies conducted at Duke University Medical School were conducted using protocols approved by the Duke Institutional Animal Care and Use Committee (IACUC) (Animal Welfare Assurance #A221-20-11) in a manner designed to minimize pain and suffering in *Mtb*-infected animals. Any animal exhibiting signs of severe disease were immediately euthanized in accordance with IACUC-approved endpoints. All mouse studies conducted at the University of Massachusetts Medical School (UMass) were performed using protocols approved by UMass IACUC (Animal Welfare Assurance #A3306-01).

### Mice

Male and female C57BL/6J (#000664) and DBA/2J (#000671) mice were purchased from The Jackson Laboratory. Male mice from 19 BXD strains were imported from the colony of Robert Williams (University of Tennessee Health Science Center, Memphis, TN, USA) in 2013. The 19 BXD strains in this study include BXD9/TyJ, BXD29/Ty, BXD39/TyJ, BXD40/TyJ, BXD48a/RwwJ, BXD51/RwwJ, BXD54/RwwJ, BXD56/RwwJ, BXD60/RwwJ, BXD62/RwwJ, BXD67/RwwJ, BXD69/RwwJ, BXD73/RwwJ, BXD73b/RwwJ, BXD77/RwwJ, BXD79/RwwJ, BXD90/RwwJ, BXD93/RwwJ, and BXD102/RwwJ. All mice were housed in a specific pathogen-free facility within standardized living conditions (12-h light/dark, food and water ad libitum). Mice were matched at 8–12 weeks of age at the time of *Mtb* infection. Male mice were used in the TnSeq screen, and both male and female mice were used in the aerosol studies.

### *Mtb* strains

All *Mtb* strains were cultured in Middlebrook 7H9 medium supplemented with oleic acid–albumin–dextrose catalase (OADC), 0.2% glycerol, and 0.05% Tween 80 to log-phase with shaking (200 rpm) at 37°C. Hygromycin (50 *µ*g/mL) and kanamycin (20 *µ*g/mL) were added when necessary. Prior to all in vivo infections, cultures were washed, resuspended in phosphate-buffered saline (PBS) containing 0.05% Tween 80 (hereafter PBS-T), and sonicated before diluting to desired concentration.

### Mouse infections

For aerosol infections, B6 and D2 were infected with ∼50 colony forming units (CFUs) of *Mtb* H37Rv YFP via aerosol inhalation (Glas-Col) for 6 and 12 weeks. At each timepoint, male and female mice were euthanized in accordance with approved IACUC protocols, and lung and spleen were harvested into PBS-T and homogenized. CFU was quantified by dilution plating onto Middlebrook 7H10 agar supplemented with OADC, 0.2% glycerol, 50 mg/mL carbenicillin, 10 mg/mL amphotericin B, 25 mg/mL polymyxin B, and 20 mg/mL trimethoprim. One lung lobe per mouse was collected into 10% neutral-buffered formalin for hematoxylin and eosin (H&E) staining by the Duke University Research Immunohistology Laboratory.

For TnSeq experiments, 1 × 10^6^ CFU of saturated *Himar1* Tn mutants (Sassetti *et al*. 2003) was delivered via intravenous tail vein injection, an infection route that enables consistent delivery of the complete TnSeq library to each mouse. Mice were infected in a single batch. At 4-week postinfection, mice were euthanized, and spleens and lungs were harvested and then homogenized in a FastPrep-24 (MP Biomedicals). CFU was quantified by dilution plating on 7H10 agar with 20 *µ*g/mL kanamycin. For library recovery, ∼1 × 10^6^ CFU per mouse was plated on 7H10 agar with 20 *µ*g/mL kanamycin. After 3 weeks of growth, colonies were harvested by scraping, and genomic DNA was extracted. The relative abundance of each Tn mutant was estimated as described in Long *et al*. (2015).

### Microscopy and damage quantification

H&E-stained lung sections were imaged in bright field at 2× magnification on the Keyence BZ-X800. Images were processed identically within FIJI software (v2.3.0/1.53p) for image clarity. To quantify damage, an artificial neural network–based damage identification model was developed within QuPath (v0.3.2) (Bankhead *et al*. 2017), as described in Wilburn *et al*. (2023). Eight images (1 image per experimental group), containing a total of 68 unaffected and 65 affected manual annotations, were utilized for the single purpose of training a pixel classifier, and the remaining 3 images per group were quantified using the model. To prevent bias, lung sections presented in this report were quantified as the closest to the mean damage of each experimental group.

### TnSeq analysis

TnSeq libraries were prepared and Tn insertion counts were estimated as previously described (Smith *et al*. 2022). The H37Rv genome annotation used is NCBI Reference Sequence NC_018143.2. In total, 40 independent Tn libraries were sequenced after recovery from the 19 BXD genotypes and the 2 parent strains, B6 and D2. Two independent Tn libraries were placed under selection within each host genotype. However, in the cases of 2 BXD genotypes, BXD48a and BXD51, only 1 library could be sufficiently recovered for sequencing. Within TRANSIT (DeJesus *et al*. 2015), beta-geometric correction was used to normalize insertion mutant counts across all libraries, and pseudocounts were implemented. Insertion counts were totaled for each *Mtb* gene, and counts were averaged between individual replicate libraries per mouse. Per gene mean values were compared with the grand mean and then log_2_ transformed.

To control for multiple hypotheses genome wide, 10,000 permutation tests were performed to generate a resampling distribution used to derive the significance of Tn mutant selection between in vitro and in vivo conditions. An *Mtb* gene was considered “essential” within a host if mutants lacking that gene experienced a log_2_ fold change (LFC) value of −0.5 or lower postselection in that host. To identify *Mtb* genes whose fitness was sufficiently variable to enable QTL mapping, the dynamic range in LFC across the parental and BXD strains for any given bacterial gene had to be at least 0.63. This value is the minimum LFC range of a mutant that was significantly selected in at least 1 host genotype after resampling (*Q* < 0.05). Mutants used for QTL mapping also needed to be sufficiently represented in the library, which was accomplished by requiring 4 or more TA Tn insertion sites to be detected for each bacterial gene. Further, each gene included needed to be significantly selected in at least 1 host genotype before resampling (*P* < 0.05).

### Genotyping and QTL mapping

Previously published BXD genotypes containing 7,321 total markers were leveraged for QTL mapping (Wang *et al*. 2016). Markers that were not diagnostic of genetic differences between B6 and D2 were filtered out resulting in a total of 7,314 markers. Genotype data and phenotype data, including lung and spleen burden and TnSeq mutant fitness profiles, were imported into R statistical software (version 4.1.1) and formatted for QTL mapping within R/qtl2 (version 0.28) (Broman *et al*. 2019). The leave-one-chromosome-out (LOCO) approach was leveraged to estimate kinship between strains as a covariate for QTL mapping. Because all screened mice were male and infected in 1 batch, no other covariates were used for the initial mapping. Logarithm of odds (LOD) scores were calculated using the linear mixed model in R/qtl2 to establish phenotypic associations with marker loci across the host genome. Trait-wise significance thresholds for QTL were established by 10,000 permutation tests. The significance of gene class overrepresentation among the mapped QTL in comparison with class representation in the whole *Mtb* genome was established using Fisher's exact test. Chromosomal ideograms of QTL across the mouse genome were generated using R/karyoploteR (Gel and Serra 2017).

## Results

### B6 and D2 mouse strains experience distinct chronic disease profiles after aerosol infection

**Fig. 1.**
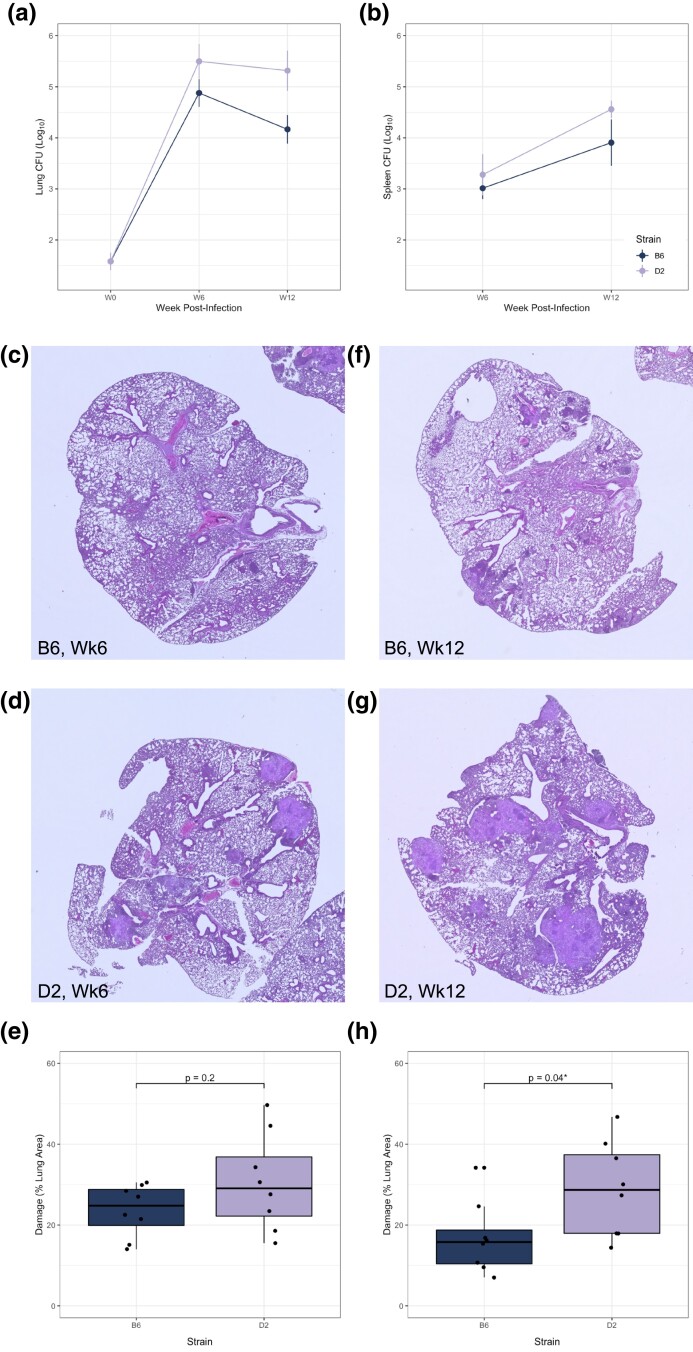
B6 and D2 are phenodeviant in TB susceptibility. a) Log_10_ transformed CFUs recovered from lung tissue throughout the course of the aerosol infection (with an infectious dose of ∼40 CFU of *Mtb* H37Rv). b) Log_10_ transformed CFU recovered from spleen throughout infection. c and d) Male B6 and D2 H&E-stained lung sections taken 6-week postinfection, 2× magnification, representative of *n* = 4 per strain. Corresponding female sections can be found in Supplementary Fig. 1c and d. e) Lung damage was quantified by QuPath v0.3.2 using an artificial neural network–based damage identification model and reported as a percent of total lung section area (*n* = 4 per genotype and sex). Lung damage is not significantly different between B6 and D2 at 6-week postinfection by unpaired Student's *t*-test. f and g) Male B6 and D2 H&E-stained lung sections taken 12-week postinfection, 2× magnification, representative of *n* = 4 per strain. Corresponding female sections can be found in Supplementary Fig. 1e and f. h) Lung damage, quantified as in e), is significantly different between B6 and D2 at 12-week postinfection by unpaired Student's *t*-test.

B6 and D2, the parent strains of the BXD panel, have divergent responses to *Mtb* infection (Chackerian and Behar 2003). Canonically considered *Mtb* resistant, the T_H_1-skewed adaptive immune response initiated by B6 mice is capable of restraining *Mtb* growth after 25 days, resulting in a mean survival time of ∼230 days (Mitsos *et al*. 2000). D2 mice control early infection with *Mtb* equally as well as B6 mice until ∼25 days. However, D2 mice experience low IFN-γ-producing T-cell influx to the lung when adaptive immunity should begin, at which point B6 and D2 begin to diverge in bacterial growth restriction (Fig. 1a and b) and lung damage (Fig. 1c–e) (Marquis *et al*. 2008). D2 macrophages become foamy and dysfunctional, making it difficult to contain and control bacterial growth in the lung (Chackerian and Behar 2003). By 12-week postaerosol infection, D2 lungs exhibit hyperinflammatory and fibrotic responses leading to overt damage (Fig. 1f–h) and an ultimate mean survival time of ∼110 days (Medina and North 1998; Keller *et al*. 2006). In addition to genotype-specific differences between these BXD parent strains, we also observe a sex effect independent of genotype, demonstrated by higher susceptibility of males of both genotypes (Supplementary Fig. 1a and b) (Tsuyuguchi *et al*. 2001; Dibbern *et al*. 2017). Further, we find that in contrast to burden, all groups reduce lung damage by week 12 except for D2 males (Supplementary Fig. 1c–g).

### Early clinical macrophenotypes fail to distinguish parental disease outcomes

**Fig. 2.**
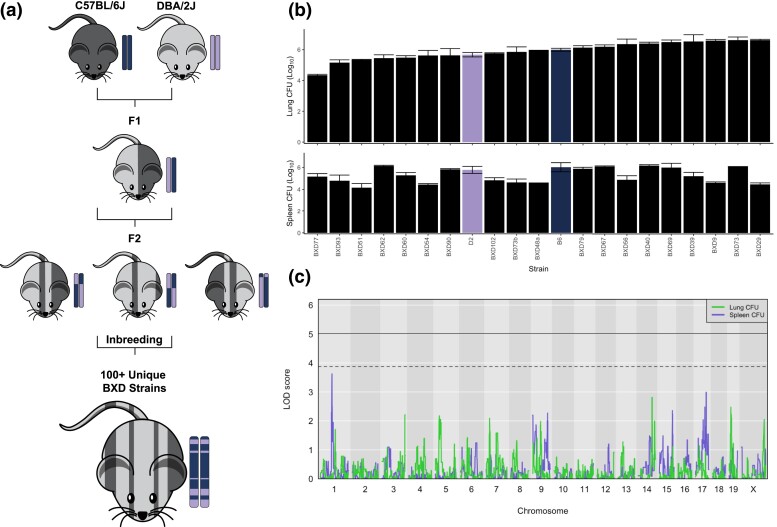
BXD panel exhibits natural genetic variation, but classical clinical traits cannot map genome-wide QTL at 4-week postinfection. a) The BXD panel is a biparental recombinant inbred panel bred from C57BL/6J (B6) and DBA/2J (D2) and composed of over 100 mosaic strains. b) Lung and spleen burden by host genotype (*n* = 1–5 per genotype). c) QTL mapping of lung and spleen burden traits across the BXD cohort. Solid threshold represents a genome-wide significance of *P* = 0.05. Dashed threshold represents *P* = 0.20. Thresholds were calculated from 10,000 permutation tests.

With over 100 still extant recombinant inbred strains bred from B6 and D2 parental strains, the BXD family is a well-suited mammalian resource to map the phenotypic diversion during *Mtb* infection (Fig. 2a). To study early disease traits and bacterial endophenotypes that predict differences in TB disease outcomes during chronic infection stages, we intravenously infected the parental B6 and D2 lines and 19 BXD genotypes with a saturated TnSeq library of Tn mutants. The library contains knockouts of every *Mtb* gene that is not required in vitro, which collectively produces an infection similar to the wild-type infection strain (Sassetti and Rubin 2003; Smith *et al*. 2022). At 4-week postinfection, the parental B6 and D2 strains demonstrated approximately the same bacterial burden in lung and spleen (Fig. 2b). Moreover, BXD strains did not exhibit sufficiently wide variation in bacterial burden at 4-week postinfection to precisely map the genetic cause of such a complex trait (Fig. 2c; Supplementary Fig. 2a and b). Compound macrophenotypes, such as CFUs in organ homogenate, are highly polygenic, making it difficult to identify causal genes even with large panels of mice (Lavebratt *et al*. 1999; Mitsos *et al*. 2000; Mitsos *et al*. 2003; Yan *et al*. 2006).

### Tn mutant fitness endophenotypes report on the host immunological microenvironment

**Fig. 3.**
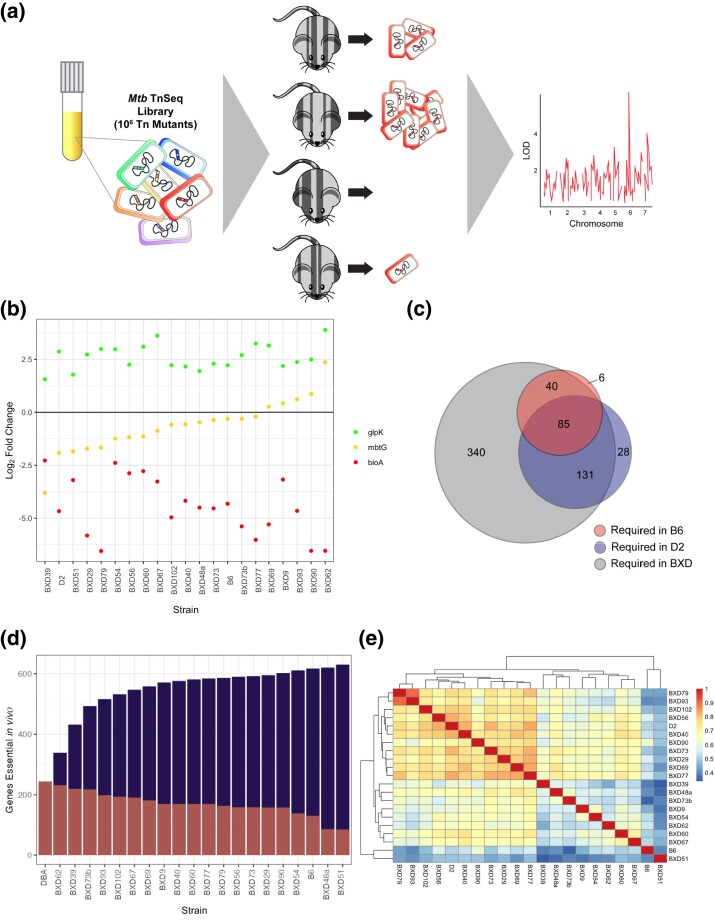
TnSeq mutant fitness is a sensitive reporter of the factors that drive disease outcome. a) Each BXD and parental strain was infected with 10^6^ CFU of *Mtb* TnSeq library, representative of ∼10^5^ independent *Mtb* Tn mutants. For each mutant, the change between the initial abundance in the TnSeq library and the abundance recovered after 4 weeks of in vivo infection was compared with calculate a quantitative fitness metric (LFC), which could be used downstream for QTL mapping. b) Example plot showing an essential *Mtb* gene (*bioA*), a conditionally essential gene (*mbtG*), and a nonessential gene (*glpK*) across the BXD panel. c) A Venn diagram depicting the overlap of essential genes between B6, D2, and the screened BXD strains. *Mtb* genes were deemed essential if the Tn mutant experienced a 1-fold reduction (LFC < −0.5) within a given host strain and were significantly different from in vitro conditions (*P* < 0.05) in at least 1 host strain across the panel. d) The number of *Mtb* genes essential (LFC < −0.5) for growth or survival in each diverse mouse strain across the panel (*P* < 0.05). Salmon (smallest circle) indicates the mutants uniquely required for each host additional strain, and purple (medium circle) shows the cumulative requirement as each new host strain is added. e) A heatmap depicting the total correlation of the library-wide bacterial fitness within each screened host strain.

To interrogate the biological mechanisms that predict immunodivergent outcomes, we quantified the abundance of each bacterial mutant both before infection with the TnSeq library and after recovery from mouse organs of each genotype, using the LFC in abundance as a quantification of relative mutant fitness in each condition. This LFC fitness value serves as a precise reporter of the host microenvironment and can be used to map host loci that impact *Mtb* mutant fitness (Fig. 3a).

We identified 3 main classes of bacterial mutants, which are highlighted in Fig. 3b. The first of these classes includes canonical *Mtb* genes that are essential for in vivo survival, exemplified by *bioA*. These genes remain essential for *Mtb* survival in both parental backgrounds and across the BXD strains (Fig. 3b; “essential” profile in red). A second, “differentially required” set of mutants is exemplified in this figure by *mbtG*; the loss of *mbtG* did not significantly reduce bacterial fitness within B6 mice, but *mbtG* mutants experienced an extreme fitness reduction within D2 mice (Fig. 3b; “differentially required” profile in yellow). Further, there is a spectrum of *mbtG* essentiality across the family, indicating that some variable host factor could modify the fitness of this mutant. In addition to in vivo essential and differentially essential *Mtb* genes, a third class of *Mtb* genes proved to be broadly dispensable for *Mtb* survival in these hosts, imparting an adaptive benefit for *Mtb* when the gene was disturbed, represented here by *glpK* (Fig. 3b; “nonessential” profile in green).

When surveying the complete set of *Mtb* Tn mutants, we find that in comparison with B6 infection, *Mtb* requires almost twice as many genes to survive within D2 mice (Fig. 3c). This finding suggests that there are detectable immunological pressures being exerted on *Mtb* within the highly inflammatory D2 line prior to the divergence in B6 and D2 bacterial control and disease outcome. This result replicates findings from previous work in which a greater number of *Mtb* genes were essential in T-cell–deficient and IFN-γ–deficient mice than in B6 (Mishra *et al*. 2017; Smith *et al*. 2022). We additionally find that the BXD panel, while recapitulating most B6- and D2-essential genes, reveals additional subsets of genes that are only required within specific BXD strains (Fig. 3d), highlighting the power of this genetically diverse panel to offer novel insights into the biology controlling TB outcome.

This spectrum of *Mtb* gene essentiality is further confirmed when we examine the correlation of the complete TnSeq library fitness between hosts (Fig. 3e). Interestingly, the majority of the strains hierarchically clustered closer to D2 than to B6, indicating that they could be inheriting factors from D2 that put greater immunological pressure on *Mtb*.

### Tn mutant fitness profiles map *hp*QTL

**Fig. 4.**
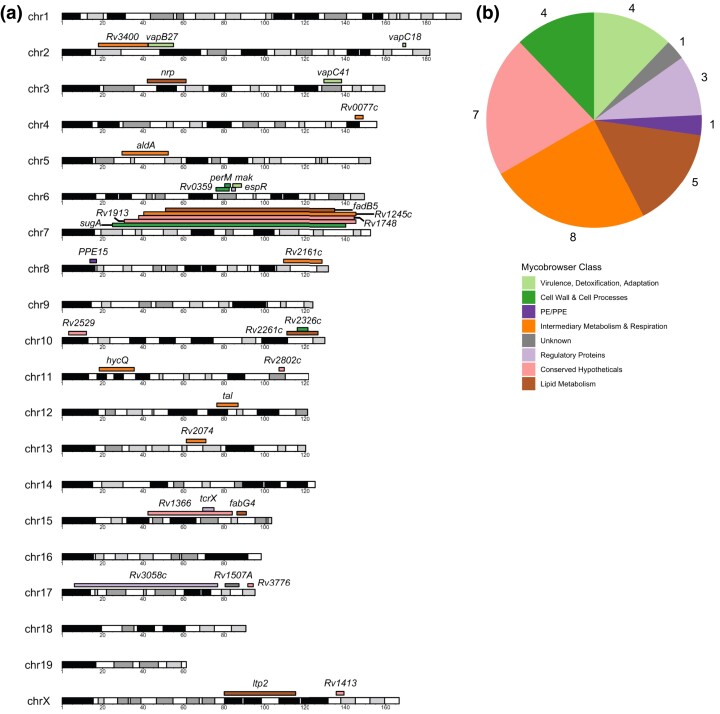
TnSeq mutant fitness endophenotypes map genome-wide significant QTL. a) The QTL that reach a significance threshold of *P* ≤ 0.01. The color of the QTL corresponds to the Mycobrowser class of the gene absent from the Tn mutant that mapped the QTL. The width of each segment corresponds to the size of the 95% Bayesian confidence interval of the QTL as calculated by R/qtl2. b) A pie chart tabulating the Mycobrowser classes of the mutants that mapped highly significant QTL (*P* ≤ 0.01), beyond the genome-wide significance threshold of *P* ≤ 0.05.

We aimed to use these bacterial mutants as sensitive reporters of host immunological pressures within the early phase of infection at the onset of adaptive immunity. Using the LFC values collected for each Tn mutant population in the TnSeq library as quantitative endophenotypes (Supplementary Table 1), we conducted QTL mapping. After removing nondynamic genes for quality control (Supplementary Fig. 3a; *Mtb* genes that are not sufficiently varying in essentiality across the screened BXD genotypes), we found 140 genome-wide significant QTL, defined as *P* ≤ 0.05 after 10,000 permutations (Table 1). Of these QTL, 33 of the loci had a *P*-value of ≤0.01 (Fig. 4a).

**Table 1.** Genome-wide significant QTL (*P* ≤ 0.05).

*hp*QTL	Trait	Gene	Chr	Position (Mb)	LOD	Start (Mb)	End (Mb)	*P*-value
hp001	Rv0913c	*Rv0913c*	1	22.773	3.52	5.022	73.222	2.24E-02
hp002	Rv0778	*cyp126*	1	121.148	3.98	115.474	126.340	1.67E-02
hp003	Rv2468c	*Rv2468c*	1	152.965	4.54	147.055	166.299	2.33E-02
hp004	Rv1260	*Rv1260*	1	155.701	4.29	143.711	160.273	3.94E-02
hp005	Rv0103c	*ctpB*	1	189.196	3.09	3.010	195.308	4.60E-02
hp006	Rv1525	*wbbL2*	2	10.808	3.56	5.653	14.395	3.32E-02
hp007	Rv3400	*Rv3400*	2	24.076	4.12	17.898	42.375	8.38E-03
hp008	Rv0625c	*Rv0625c*	2	45.533	3.37	25.645	161.538	5.01E-02
hp009	Rv0599c	*vapB27*	2	46.212	3.74	42.557	54.975	7.86E-03
hp010	Rv3294c	*Rv3294c*	2	138.850	3.56	136.128	158.741	3.73E-02
hp011	Rv3565	*aspB*	2	159.316	3.88	42.557	174.820	1.47E-02
hp012	Rv2546	*vapC18*	2	169.114	4.90	168.446	169.887	1.80E-03
hp013	Rv1965	*yrbE3B*	2	170.362	3.50	15.435	180.835	1.43E-02
hp014	Rv1367c	*Rv1367c*	2	173.385	3.77	46.133	176.951	1.85E-02
hp015	Rv1290A	*Rv1290A*	2	181.009	2.96	14.698	181.991	2.36E-02
hp016	Rv3047c	*Rv3047c*	3	41.212	3.92	39.644	51.561	4.49E-02
hp017	Rv0101	*nrp*	3	57.642	3.69	42.035	61.219	6.19E-03
hp018	Rv2051c	*ppm1*	3	62.392	4.25	61.219	70.185	1.94E-02
hp019	Rv1935c	*echA13*	3	129.496	4.23	10.997	157.792	4.20E-02
hp020	Rv1735c	*Rv1735c*	3	129.508	3.64	117.504	157.140	4.20E-02
hp021	Rv2602	*vapC41*	3	132.174	4.01	129.509	138.245	1.12E-02
hp022	Rv0773c	*ggtA*	3	146.239	4.56	143.570	156.034	2.75E-02
hp023	Rv0077c	*Rv0077c*	4	144.942	4.31	144.913	148.864	1.04E-02
hp024	Rv0588	*yrbE2B*	4	153.793	3.76	54.951	155.498	3.23E-02
hp025	Rv0093c	*Rv0093c*	5	28.411	3.05	19.137	132.038	2.29E-02
hp026	Rv0768	*aldA*	5	43.085	4.19	29.537	52.408	3.09E-03
hp027	Rv2957	*Rv2957*	5	117.550	3.27	3.143	139.501	3.26E-02
hp028	Rv3848	*Rv3848*	5	136.015	5.69	133.911	138.657	1.55E-02
hp029	Rv0359	*rip2*	6	76.492	4.18	75.970	82.500	5.63E-03
hp030	Rv0955	*perM*	6	82.500	4.80	80.286	83.092	1.29E-02
hp031	Rv2140c	*TB18.6*	6	84.277	4.62	80.286	85.650	2.28E-02
hp032	Rv3849	*espR*	6	84.869	6.77	83.684	85.566	6.23E-03
hp033	Rv0127	*mak*	6	85.650	7.83	84.277	88.578	7.04E-03
hp034	Rv1056	*Rv1056*	6	93.600	4.10	89.371	95.127	4.40E-02
hp035	Rv2671	*ribD*	6	148.818	3.53	12.797	149.266	1.60E−02
hp036	Rv3711c	*dnaQ*	7	27.300	3.31	24.735	145.321	3.80E−02
hp037	Rv3299c	*atsB*	7	36.692	3.01	3.078	144.446	5.34E−02
hp038	Rv0065	*vapC1*	7	37.008	3.41	3.078	144.445	4.72E−02
hp039	Rv1530	*adh*	7	37.008	3.22	3.078	145.321	3.74E−02
hp040	Rv2482c	*plsB2*	7	37.008	3.55	24.619	144.445	2.35E−02
hp041	Rv0026	*Rv0026*	7	37.760	3.34	24.764	128.756	2.82E−02
hp042	Rv1913	*Rv1913*	7	39.822	3.63	30.689	145.321	2.96E−03
hp043	Rv1236	*sugA*	7	40.289	3.13	24.822	140.297	5.28E−03
hp044	Rv1528c	*papA4*	7	40.289	4.97	40.226	46.357	2.11E−02
hp045	Rv1542c	*glbN*	7	40.289	3.71	3.078	145.321	5.77E−02
hp046	Rv1992c	*ctpG*	7	40.289	3.24	6.015	145.321	3.20E−02
hp047	Rv3702c	*Rv3702c*	7	40.289	4.55	24.793	140.297	2.75E−02
hp048	Rv2388c	*hemN*	7	84.014	3.39	39.894	127.779	2.54E−02
hp049	Rv0852	*fadD16*	7	97.465	3.71	83.879	128.058	3.89E−02
hp050	Rv0878c	*PPE13*	7	97.685	4.10	89.870	127.500	2.49E−02
hp051	Rv0089	*Rv0089*	7	116.709	4.08	98.124	127.989	3.64E−02
hp052	Rv1912c	*fadB5*	7	134.484	4.49	51.060	134.831	1.14E−02
hp053	Rv3868	*eccA1*	7	134.598	3.80	17.136	144.445	4.40E−02
hp054	Rv2637	*dedA*	7	140.297	4.63	138.137	140.586	1.63E−02
hp055	Rv0271c	*fadE6*	7	140.586	5.74	140.586	143.684	3.53E−02
hp056	Rv1984c	*cfp21*	7	140.586	3.44	24.938	144.445	1.68E−02
hp057	Rv1104	*Rv1104*	7	143.809	3.29	24.735	145.321	2.49E−02
hp058	Rv3179	*Rv3179*	7	143.809	3.78	140.586	144.445	5.34E−02
hp059	Rv1748	*Rv1748*	7	144.184	5.42	37.760	144.446	7.25E−03
hp060	Rv3288c	*usfY*	7	144.184	3.97	30.593	145.321	3.46E−02
hp061	Rv3698	*Rv3698*	7	144.309	3.41	12.026	145.321	3.60E−02
hp062	Rv1245c	*Rv1245c*	7	144.446	3.97	40.289	145.321	2.62E−03
hp063	Rv2092c	*helY*	7	144.687	5.34	144.533	145.321	2.50E−02
hp064	Rv0458	*Rv0458*	7	145.321	3.92	144.448	145.321	2.93E−02
hp065	Rv0538	*Rv0538*	8	11.279	4.57	11.279	13.680	1.47E−02
hp066	Rv2066	*cobI*	8	14.819	4.31	12.880	15.343	4.00E−02
hp067	Rv1039c	*PPE15*	8	16.687	4.55	13.680	16.946	1.31E−02
hp068	Rv3855	*ethR*	8	87.897	4.62	87.219	89.367	1.44E−02
hp069	Rv2161c	*Rv2161c*	8	113.708	4.09	109.407	128.590	5.93E−03
hp070	Rv2384	*mbtA*	9	29.939	4.02	4.359	107.777	3.24E−02
hp071	Rv1954c	*Rv1954c*	9	34.618	4.70	29.941	58.854	2.24E−02
hp072	Rv3899c	*Rv3899c*	9	94.905	4.16	87.462	101.324	3.61E−02
hp073	Rv0950c	*Rv0950c*	9	101.365	3.92	4.359	106.019	2.64E−02
hp074	Rv0538	*Rv0538*	9	103.686	4.05	80.003	106.019	4.83E−02
hp075	Rv3087	*Rv3087*	10	5.031	4.52	3.181	9.092	3.38E−02
hp076	Rv2529	*Rv2529*	10	9.413	4.33	3.181	11.991	9.41E−03
hp077	Rv1450c	*PE_PGRS27*	10	19.780	4.04	13.853	21.509	3.92E−02
hp078	Rv1507c	*Rv1507c*	10	55.483	4.97	55.356	59.293	1.73E−02
hp079	Rv1366	*Rv1366*	10	64.014	3.25	57.276	72.506	2.31E−02
hp080	Rv1552	*frdA*	10	96.749	3.97	95.639	103.562	3.45E−02
hp081	Rv1753c	*PPE24*	10	109.165	3.52	13.688	127.327	1.75E−02
hp082	Rv2261c	*Rv2261c*	10	111.074	4.75	110.998	126.607	1.16E−02
hp083	Rv0044c	*Rv0044c*	10	115.897	3.42	3.181	127.808	6.21E−02
hp084	Rv2326c	*Rv2326c*	10	120.610	4.29	116.134	121.419	1.26E−02
hp085	Rv0769	*Rv0769*	10	126.740	4.12	123.469	126.808	2.36E−02
hp086	Rv2993c	*Rv2993c*	11	11.620	4.35	8.814	15.810	3.65E−02
hp087	Rv3759c	*proX*	11	21.073	4.70	19.834	34.416	4.71E−02
hp088	Rv0086	*hycQ*	11	24.850	3.37	18.284	35.583	1.22E−02
hp089	Rv3841	*bfrB*	11	47.049	3.28	35.903	80.586	1.67E−02
hp090	Rv0126	*treS*	11	65.541	3.56	64.895	81.165	3.85E−02
hp091	Rv2116	*lppK*	11	73.243	4.46	73.194	81.165	1.67E−02
hp092	Rv0765c	*Rv0765c*	11	106.661	4.70	106.040	107.457	2.58E−02
hp093	Rv1850	*ureC*	11	107.742	4.25	107.422	110.228	1.61E−02
hp094	Rv2802c	*Rv2802c*	11	107.742	4.87	107.133	109.751	9.90E−03
hp095	Rv1452c	*PE_PGRS28*	11	107.964	3.29	103.707	111.341	4.10E−02
hp096	Rv0599c	*vapB27*	11	113.557	3.58	110.546	118.682	1.76E−02
hp097	Rv1969	*mce3D*	12	46.628	4.14	27.823	52.908	4.09E−02
hp098	Rv3554	*fdxB*	12	71.873	3.22	58.970	114.110	4.64E−02
hp099	Rv1448c	*tal*	12	79.511	4.90	76.378	86.909	1.38E−02
hp100	Rv2922c	*smc*	12	105.499	4.57	104.281	107.191	3.70E−02
hp101	Rv2268c	*cyp128*	12	107.369	3.62	5.594	107.848	3.59E−02
hp102	Rv1775	*Rv1775*	12	108.105	3.71	107.369	108.898	3.33E−02
hp103	Rv3164c	*moxR3*	12	114.166	4.13	111.180	116.685	3.60E−02
hp104	Rv2019	*Rv2019*	13	6.304	3.82	4.432	115.537	4.09E−02
hp105	Rv2379c	*mbtF*	13	18.035	3.66	4.432	45.238	3.69E−02
hp106	Rv0066c	*icd2*	13	39.372	4.40	34.110	39.515	1.60E−02
hp107	Rv1250	*Rv1250*	13	50.000	3.82	45.902	54.614	2.04E−02
hp108	Rv2282c	*Rv2282c*	13	54.862	3.93	54.843	72.958	3.30E−02
hp109	Rv2074	*Rv2074*	13	63.704	5.16	61.332	71.002	6.80E−03
hp110	Rv0552	*Rv0552*	13	67.497	5.30	63.792	67.497	3.26E−02
hp111	Rv1009	*rpfB*	13	75.762	4.75	72.969	84.394	2.23E−02
hp112	Rv0223c	*Rv0223c*	14	73.646	4.05	73.198	79.374	2.12E−02
hp113	Rv3063	*cstA*	14	79.403	3.63	61.301	118.199	1.55E−02
hp114	Rv1525	*wbbL2*	15	27.520	3.75	11.145	102.347	1.79E−02
hp115	Rv3013	*Rv3013*	15	28.265	3.98	12.673	28.312	1.65E−02
hp116	Rv3354	*Rv3354*	15	28.312	2.83	9.237	102.347	4.35E−02
hp117	Rv1664	*pks9*	15	32.262	2.86	7.168	72.449	3.57E−02
hp118	Rv1366	*Rv1366*	15	64.513	3.43	42.380	84.022	9.72E−03
hp119	Rv3765c	*tcrX*	15	74.502	4.19	69.446	75.001	6.29E−03
hp120	Rv0454	*Rv0454*	15	75.750	4.13	75.001	82.088	1.87E−02
hp121	Rv2315c	*Rv2315c*	15	84.037	3.37	7.814	102.354	4.37E−02
hp122	Rv0242c	*fabG4*	15	88.975	5.10	86.329	90.948	5.91E−03
hp123	Rv0959	*Rv0959*	15	91.863	4.47	90.948	93.408	4.13E−02
hp124	Rv1961	*Rv1961*	15	95.169	4.46	94.529	96.191	5.17E−02
hp125	Rv0043c	*Rv0043c*	16	44.080	3.98	36.743	44.128	2.51E−02
hp126	Rv1400c	*lipI*	16	78.088	3.52	75.905	85.637	3.92E−02
hp127	Rv1552	*frdA*	16	92.557	3.95	74.904	96.503	3.60E−02
hp128	Rv3058c	*Rv3058c*	17	55.278	4.32	6.007	76.812	2.40E−04
hp129	Rv1507A	*Rv1507A*	17	85.527	4.44	80.477	87.364	6.91E−03
hp130	Rv3776	*Rv3776*	17	94.132	4.63	91.701	94.411	1.16E−02
hp131	Rv3495c	*lprN*	18	57.375	4.87	57.348	58.270	3.71E−02
hp132	Rv1084	*Rv1084*	19	10.714	4.90	3.337	14.209	2.73E−02
hp133	Rv2378c	*mbtG*	19	23.841	3.02	21.915	27.750	3.72E−02
hp134	Rv0056	*rplI*	19	39.978	4.00	38.651	42.427	3.83E−02
hp135	Rv3540c	*ltp2*	X	101.772	4.43	80.056	115.391	3.14E−03
hp136	Rv0694	*lldD1*	X	115.365	3.91	102.867	167.395	3.70E−02
hp137	Rv1791	*PE19*	X	119.825	4.38	115.497	128.620	2.38E−02
hp138	Rv2733c	*Rv2733c*	X	133.591	3.40	115.471	138.012	3.90E−02
hp139	Rv0182c	*sigG*	X	136.470	4.68	136.304	138.007	1.61E−02
hp140	Rv1413	*Rv1413*	X	138.012	4.90	135.586	139.435	5.84E−03

“*hp*QTL” is the name for each host-pathogen QTL of genome-wide significance (*P* ≤ 0.05). “Trait” refers to the Rv number of the bacterial mutant whose fitness profile identified the *hp*QTL in the host genome. “Gene” is the bacterial gene annotation of the mutant if one is available on Mycobrowser. “Chr” is the host chromosome on which the QTL is located, and “Position (Mb)” is the position of the QTL in megabases. “LOD” is the maximum association score between the mutant fitness profile and the host locus. “Start (Mb)” and “End (Mb)” are the lower and upper boundaries of the Bayesian 95% confidence interval, as calculated by R/qtl2. “*P*-value” denotes the significance of the association between the bacterial mutant fitness and the host genetic locus.

Among the most significant QTL mapped in the screen, certain gene classes were marginally overrepresented in comparison with the genome-wide abundance of that class. Tn mutants lacking genes that encode known virulence factors made up 12.1% of the top hits in this screen despite only representing 5.8% of the *Mtb* genome (*P* = 0.1) (Fig. 4b) (Kapopoulou *et al*. 2011). Further, lipid metabolism genes made up 15.2% of the bacterial mutants mapping highly significant QTL while representing 6.6% of the *Mtb* genome (*P* = 0.07). Certain gene classes were abundant in approximately equal proportions in the mapped QTL as they were genome wide, such as metabolism and respiration genes (22.8% among top QTL vs 24.2% genome-wide prevalence) and genes encoding PPE/PE proteins (3.0% among top QTL vs 4.1% genome-wide prevalence). Roughly half of *Mtb* protein-coding genes remain unannotated (Whitaker *et al*. 2020), and 7 of the highly significant (*P* ≤ 0.01) bacterial fitness traits mapped by this screen correspond to bacterial genes with no known function. Taking these screen data in combination with previous in vivo TnSeq studies, these data will contribute to deorphaning the *Mtb* genome.

### Multiple highly significant QTL map to host chromosome 6 “hotspot”

**Fig. 5.**
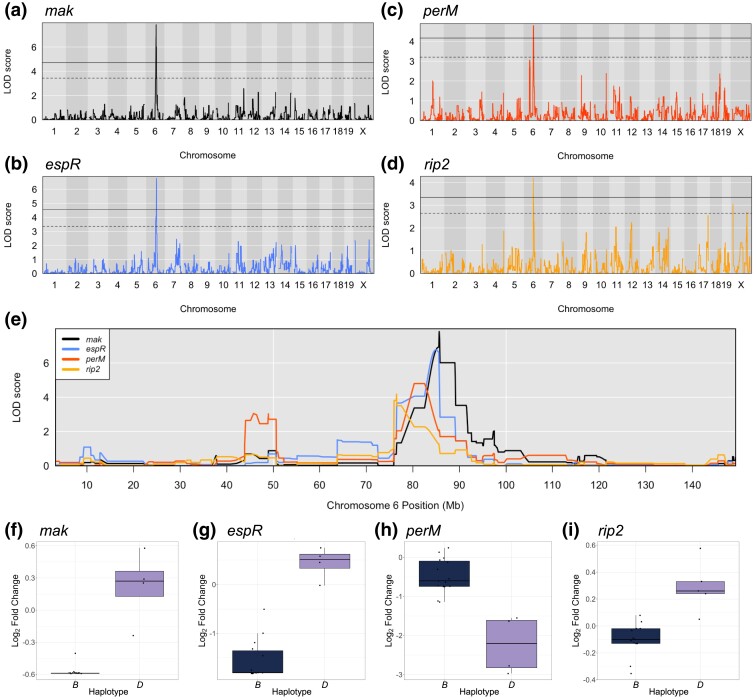
A chromosome 6 QTL hotspot highlights a genomic region controlling fitness of multiple bacterial mutants. QTL mapping of Tn mutants lacking a) *mak*, b) *espR*, c) *perM*, and d) *rip2*. After 10,000 permutation tests, the solid threshold represents *P* = 0.05, and the dashed threshold represents *P* = 0.20. e) Chromosome 6 mapping overlap of the 4 Tn mutant fitness profiles that identified the QTL hotspot. f–i) Boxplots representing the fitness of Tn mutants lacking *mak*, *espR*, *perM*, and *rip2* within BXD mice with *B* (navy) or *D* (lilac) haplotypes at the QTL position. BXD genotypes for which a haplotype state could not be called with 95% confidence were not included.

The nature of this screen enabled the discovery of host loci that impact the fitness of numerous bacterial mutants. Among the QTL mapped in this screen, 5 QTL mapped to chromosome 7 (Fig. 4a), yet the 95% confidence intervals for each QTL span such a large part of the chromosome that identifying a causal host gene would be infeasible without further experimentation. Aside from the chromosome 7 locus, there appears to be a much narrower region on chromosome 6 (75.97–88.58 Mb) strongly linked to the essentiality of 4 bacterial genes: *Rv0127* (*mak*; sometimes annotated as *pep2*), *Rv0359* (*rip2*), *Rv0955* (*perM*), and *Rv3849* (*espR*) (Fig. 5a–e). For each of the mutants lacking these bacterial genes, apart from *perM*, the B6 sequence in this region is associated with lower bacterial mutant fitness (Fig. 5f–i; Supplementary Fig. 4a–h). *Mtb* mutants lacking *perM* experience a fitness cost within genotypes that possess the *D* haplotype in this region.

This QTL hotspot is mapped by *Mtb* genes that impact metabolism, virulence, and defense. Mak catalyzes the ATP-dependent conversion of maltose into maltose-1-phosphate as a part of the glycogen biosynthesis pathway that supports construction of the protective outer capsule of *Mtb* (Li *et al*. 2014). Of note, TreS, which converts trehalose to maltose upstream of Mak, also maps to a genome-wide significant QTL on chromosome 11 and has done so in previous *Mtb* infection screens (Smith *et al*. 2022). *rip2* is a putative zinc metalloprotease located in the *Mtb* cell membrane (Sklar *et al*. 2010), although very little is known about its function. PerM is an integral membrane protein that is known to play an essential role in enabling bacterial cell division under acid stress in B6 mice (Wang *et al*. 2019). Here, we report that *perM* mutants are even more severely attenuated within a D2 background, implying that PerM is differentially impacted by host immunological pressures. EspR is a key regulator of the ESX-1 secretion system (Rosenberg *et al*. 2011; Blasco *et al*. 2012), which itself plays a well-established role in mycobacterial virulence. Mutants lacking these bacterial genes map significant QTL within the interval of 75.97–88.58 Mb on mouse chromosome 6.

**Fig. 6.**
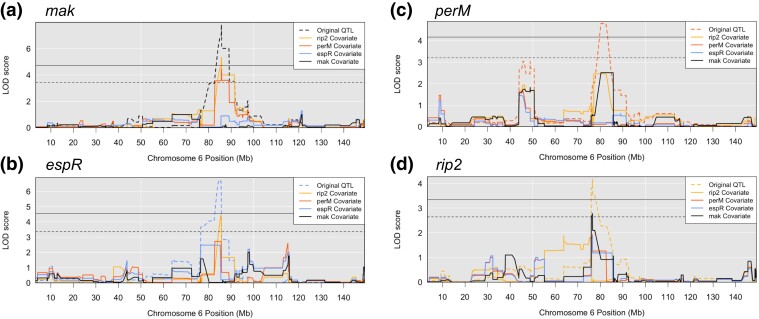
Independence tests identify 2 putative causal variants. Scan2 independence tests from R/qtl were used to identify whether more than 1 causal host factor may underlie the chromosome 6 hotspot. The QTL mapped by a) *mak*, b) *espR*, c) *perM*, and d) *rip2* Tn mutants were remapped, incorporating the haplotype of the BXD mice at the locus of each other QTL as a covariate in the mapping analysis. The original QTL mapped by each mutant fitness profile are represented as dashed lines. The solid horizontal threshold represents *P* = 0.05 while the dashed horizontal threshold represents *P* = 0.20.

The 95% Bayesian confidence intervals of the QTL mapped by *rip2* and *perM* mutants overlap while the confidence intervals of *mak* and *espR* mutants overlap separately from the *rip2* and *perM* QTL. To test for the independence of these 2 groups of nearby QTL, we remapped each of the QTL within the chromosome 6 hotspot, iteratively incorporating the haplotype possessed by each BXD strain at each QTL as an additive covariate. Dependent loci will not map if the haplotype of 1 locus is factored into the mapping pipeline as a covariate. Conversely, if a QTL achieves significance by permutation test while the haplotype state at another significant QTL nearby is factored into mapping as a covariate, it suggests that the 2 loci arise from distinct causal genetic factors within the host. These Scan2 independence tests, so named in R/qtl, suggested 2 causal host loci exist within this highly significant QTL hotspot: 1 host factor underlying the *rip2* and *perM* mutant fitness QTL and 1 underlying the *mak* and *espR* QTL (Fig. 6a–d).

**Fig. 7.**
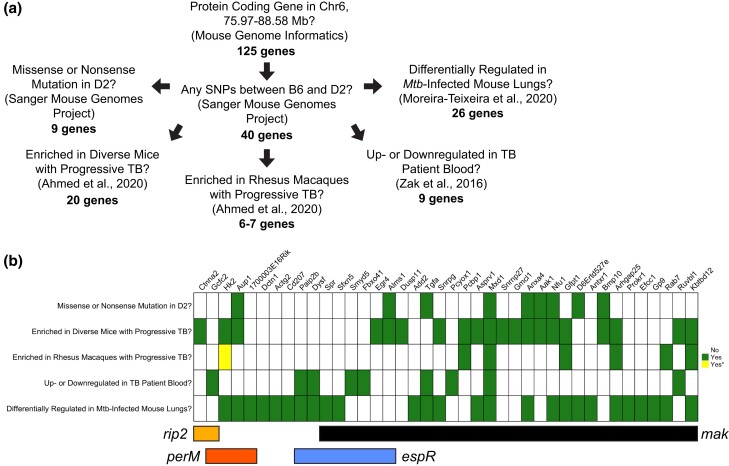
A bioinformatic pipeline highlights putative candidate genes underlying the QTL on chromosome 6. a) A graphic summarizing the bioinformatic pipeline used to prioritize host gene candidates within each QTL. b) A heatmap representative of the per-gene outcome of 5 distinct criteria: (1) whether or not D2 possesses a missense or nonsense mutation in comparison to B6 according to the Wellcome Sanger Institute Mouse Genomes Project (Sanger MGP) (Adams *et al*. 2015), (2) whether the gene is significantly enriched in genetically diverse mice progressively infected with *Mtb* (Ahmed *et al*. 2020), (3) whether the gene is significantly enriched in Rhesus Macaques progressively infected with *Mtb* (Ahmed *et al*. 2020), (4) whether the gene is significantly upregulated or downregulated in TB patient blood (Zak *et al*. 2016), and (5) whether the gene is differentially expressed in mouse lungs across variable host genotypes, *Mtb* genotypes, and doses (Moreira-Teixeira *et al*. 2020). If the answer is no, the space remains blank. Green indicates yes, and yellow indicates yes but cautions that the Rhesus Macaque gene is not a high confidence homolog. Only protein-coding genes with a D2 SNP (as per Sanger MGP) that met at least 1 of the criteria are included in this visual.

To identify host gene candidates that may mediate the survival of these 2 groups of bacterial mutants, we defined a pipeline to select for protein-coding genes for which *D* haplotype has a unique SNP in comparison with *B* haplotype (Fig. 7a). We passed each of the genes identified by this pipeline through a list of genetic and clinical criteria using independent but complementary data sets from a variety of *Mtb*-infected mammalian cohorts (Fig. 7b) (Zak *et al*. 2016; Moreira-Teixeira *et al*. 2017; Ahmed *et al*. 2020). From this comparative analysis, we identified a putative host candidate within the region mapped by *mak*: MAX dimerization protein 1 (*Mxd1*), which is known to be upregulated in murine bone marrow-derived macrophages after infection with the hypervirulent *Mtb* HN878 strain (Roy *et al*. 2018). Further, we found a putative host candidate within the QTL mapped by *perM* mutants: ancient ubiquitous protein 1 (*Aup1*). The QTL mapped by the *mak* Tn mutant was enriched for protein-coding genes with D2-specific SNPs compared with the other QTL within the hotspot. However, the causal locus underlying these QTL may not be protein coding at all. In fact, historical studies of cardiomyopathy in B6 and D2 backcrosses have identified a QTL that was later discovered to be caused by an intronic variant in the tropin-interacting kinase gene *Tnni3k* (Wheeler *et al*. 2005; Wheeler *et al*. 2009), which also conferred susceptibility to viral-induced myocarditis (Wiltshire *et al*. 2011). There are many examples in literature of noncoding causal variants (Hamilton and Yu 2012), yet there remain compelling candidate genes within the chromosome 6 hotspot that could drive the conditional essentiality of these *Mtb* genes in vivo. Altogether, we have leveraged complementary screen data to identify plausible host gene candidates underlying these *host–pathogen* QTL (*hp*QTL).

## Discussion

While it is thought that nearly a quarter of the global population is infected with *Mtb* (Houben and Dodd 2016), only 5–10% of *Mtb* infections ultimately result in active TB (Gopal *et al*. 2014). This paradox has motivated decades of study on human genetic variants that could predict such a lethal prognosis in affected individuals. While Genome-wide association studies (GWAS) among infected populations has identified numerous host loci associated with increased susceptibility to disease, a limited ability to dissect biological mechanism leaves many questions unanswered (Saul *et al*. 2019). QTL mapping, a parallel systems genetics technique, has been utilized for decades to identify host loci in genetically tractable populations linked to TB susceptibility; Sst1, one of the most well-known susceptibility loci, was first discovered through a QTL mapping study in mice (Kramnik *et al*. 2000). Despite the widespread use of disease traits, including mean survival time, body weight, and bacterial burden, to identify host determinants of disease progression (Lavebratt *et al*. 1999; Kramnik *et al*. 2000; Mitsos *et al*. 2000; Mitsos *et al*. 2003; Yan *et al*. 2006), these phenotypes can fail to dissect immunological features that drive distinct *Mtb* pathogenesis in diverse hosts. Here, we describe a screen in which we leveraged the reproducibility, host variation, and phenotypic divergence of the BXD family and the molecular precision of the TnSeq library to identify novel axes of host–pathogen interaction during *Mtb* infection. We demonstrate the late (12-week) phenodivergence of the parental B6 and D2 mice; however, we found that CFU serves as a weak predictor of host outcome at the onset of adaptive immunity 4-week postinfection. By utilizing TnSeq as a refined infection trait, we identified bacterial mutants that could distinguish the resistant B6 and susceptible D2 strains at the early 4-week infection timepoint. Finally, we leveraged the differentially required mutants as traits to conduct QTL mapping and identified 140 genome-wide *hp*QTL and a QTL hotspot encompassing a cluster of *Mtb* virulence and cell wall genes. Using this BXD TnSeq platform and a novel candidate prioritization pipeline, we identified viable host gene candidates for future study.

This screen highlights the importance of intentionality in design for deep phenotyping platforms to optimally identify the causal factors underlying gene associations. In contrast to complex macrophenotypes, which require hundreds of mice to sufficiently power causal locus identification for highly polygenic traits, molecular endophenotypes, such as *Mtb* Tn mutant fitness, allow us to learn more about the intricate details of *Mtb* invasion in the early stages of infection, which is particularly helpful in an experimental framework with fewer host genotypes. The strength of TnSeq as a deep phenotyping platform lies in the redundancy of each *Mtb* gene knockout; within the TnSeq library, each bacterial gene that is nonessential in vitro was perturbed at 4 or more unique Tn insertion sites, resulting in multiple subpopulations of unique replicates of each *Mtb* gene knockout. These mutant replicates taken together paint a robust picture of individual *Mtb* gene requirement in vivo, and multiple host replicates strengthen the reproducibility of each *Mtb* mutant fitness profile. In contrast with our previous TnSeq study that utilized nearly 70 host genotypes (Smith *et al*. 2022), this TnSeq study includes 21 unique genotypes and 73 mice, including the parental strains B6 and D2. The greater number of Tn mutant *hp*QTL identified in this screen demonstrates the power of a 2-state model to segregate allele effects, as opposed to the 8-state model present in the CC recombinant inbred panel. We, however, cannot eliminate the possibility that the smaller number of host genotypes included in this study did not sufficiently limit statistical noise during QTL mapping analyses.

Using bacterial mutant profiles as endophenotypes, we identified 140 genome-wide significant (*P* ≤ 0.05) associations between *Mtb* Tn mutant survival and host genetic loci in a diverse cohort, which may contribute to divergence in clinical outcomes between *Mtb*-resistant and susceptible hosts. These *hp*QTL and the Tn mutant fitness profiles across the BXD panel have been shared with GeneNetwork.org (Chesler *et al*. 2004; Parker *et al*. 2017; Ashbrook *et al*. 2021), an online compendium of BXD phenotypes. Increasing the precision of bacterial traits creates a rich environment for screen-based hypothesis generation in a smaller host population, thereby lowering the cost through lower animal count and less total infection time. This screen serves as a proof of concept that large host populations are not an absolute necessity to uncover novel and biologically meaningful axes of the host–pathogen interface. Molecular endophenotyping enables a much more accessible experimental platform to assist in our ongoing struggle against *Mtb*.

From this discovery screen, we have identified several plausible host gene candidates. Variants in *Aup1* represent a possible axis for interaction between *Mtb* and the mammalian host. The acetyltransferase activity of *Aup1* has been shown to be exploited by flaviviruses to trigger autophagy of lipid droplets within the cell, which produces ATP for the virus, thereby promoting viral replication (Zhang *et al*. 2018). Although not much is known about whether *Mtb* interacts directly with this protein, *Mtb* is one of the few bacteria capable of generating lipid droplets (Daniel *et al*. 2011). Host lipid droplets, specifically those derived from foamy macrophages, have been shown to promote the production of defensive cytokines during *Mtb* infection (Jaisinghani *et al*. 2018), implicating a potentially intriguing locus for host–pathogen interaction. The other high priority candidate *Mxd1* encodes the protein MAD, which competes with MYC to bind with MAX, forming a transcriptional repressor. This mechanism has led *Mxd1* to be considered a putative tumor suppressor gene. *Mxd1* has been shown to be upregulated in murine bone marrow–derived macrophages upon initial infection with the hypervirulent *Mtb* HN878 strain (Roy *et al*. 2018) and to regulate the fitness of murine dendritic cells (Anderson *et al*. 2020), yet the function of *Mxd1* in response to *Mtb* has yet to be determined. Together, these candidates provide feasible next steps for mechanistic interrogation of these loci.

While the functions of many mycobacterial genes remain unknown (Whitaker *et al*. 2020), this Tn mutant screen within the BXD family builds directly upon a previous screen in the CC panel (Smith *et al*. 2022) to further articulate the conditional necessity of many canonically “nonessential” *Mtb* genes within a spectrum of host microenvironments. A substantial number of *Mtb* genes known to be nonessential in B6 mice prove to be essential in a subset of these genetically diverse hosts, suggesting that these bacterial genes play an active yet undiscovered role in pathogenesis under specific immunological conditions. These new host conditions have been shared with MtbTnDB (Jinich *et al*. 2021), a central repository encompassing over 60 published *Mtb* TnSeq screens for ease of browsing and interscreen comparison. For mycobacterial researchers exploring *Mtb* genes traditionally thought to be nonessential in vivo, this work challenges such a notion and offers a new model that will provide insight into the host–pathogen dynamic underlying this conditional in vivo essentiality.

## Supplementary Material

jkad147_Supplementary_Data

## Data Availability

All relevant data to support the findings of this study are located within the paper and supplementary files. Genome sequence data is available in the NCBI Gene Expression Omnibus (GEO), accession number GSE234093. All QTL mapping files are publicly available at: https://github.com/SmithLabDuke/Meade_et_al_BXD_TnSeq. Tn mutant fitness profiles within each BXD genotype and parental genotypes are publicly available on MtbTnDB (https://www.mtbtndb.app/). BXD phenotypes are available on GeneNetwork (https://www.genenetwork.org). Supplemental material available at G3 online.

## References

[jkad147-B1] Adams DJ , Doran AG, Lilue J, Keane TM. The mouse genomes project: a repository of inbred laboratory mouse strain genomes. Mamm Genome. 2015;26(9–10):403–412. doi:10.1007/s00335-015-9579-6. 26123534

[jkad147-B2] Ahmed M , Thirunavukkarasu S, Rosa BA, Thomas KA, Das S, Rangel-Moreno J, Lu L, Mehra S, Mbandi SK, Thackray LB, et al Immune correlates of tuberculosis disease and risk translate across species. Sci Transl Med. 2020;12(528):eaay0233. . doi:10.1126/scitranslmed.aay0233. 31996462 PMC7354419

[jkad147-B3] Anderson DA III , Murphy TL, Eisenman RN, Murphy KM. The MYCL and MXD1 transcription factors regulate the fitness of murine dendritic cells. Proc Natl Acad Sci U S A. 2020;117(9):4885–4893. doi:10.1073/pnas.1915060117. 32071205 PMC7060746

[jkad147-B4] Ashbrook DG , Arends D, Prins P, Mulligan MK, Roy S, Williams EG, Lutz CM, Valenzuela A, Bohl CJ, Ingels JF, et al A platform for experimental precision medicine: the extended BXD mouse family. Cell Syst. 2021;12(3):235–247.e9. . doi:10.1016/j.cels.2020.12.002. 33472028 PMC7979527

[jkad147-B5] Ashbrook DG , Sasani T, Maksimov M, Gunturkun MH, Ma N, Villani F, Ren Y, Rothschild D, Chen H, Lu L, et al 2022. Private and sub-family specific mutations of founder haplotypes in the BXD family reveal phenotypic consequences relevant to health and disease. bioRxiv 2022.04.21.489063. 10.1101/2022.04.21.48906

[jkad147-B6] Bankhead P , Loughrey MB, Fernández JA, Dombrowski Y, McArt DG, Dunne PD, McQuaid S, Gray RT, Murray LJ, Coleman HG, et al Qupath: open source software for digital pathology image analysis. Sci Rep. 2017;7(1):16878. doi:10.1038/s41598-017-17204-5. 29203879 PMC5715110

[jkad147-B7] Bellerose MM , Baek S-H, Huang C-C, Moss CE, Koh E-I, Proulx MK, Smith CM, Baker RE, Lee JS, Eum S, et al Common variants in the glycerol kinase gene reduce tuberculosis drug efficacy. mBio. 2019;10(4):e00663-19. . doi:10.1128/mBio.00663-19. 31363023 PMC6667613

[jkad147-B8] Blasco B , Chen JM, Hartkoorn R, Sala C, Uplekar S, Rougemont J, Pojer F, Cole ST. Virulence regulator EspR of *Mycobacterium tuberculosis* is a nucleoid-associated protein. PLoS Pathog. 2012;8(3):e1002621. . doi:10.1371/journal.ppat.1002621. 22479184 PMC3315491

[jkad147-B9] Broman KW , Gatti DM, Simecek P, Furlotte NA, Prins P, Sen Ś, Yandell BS, Churchill GA. R/qtl2: software for mapping quantitative trait loci with high-dimensional data and multiparent populations. Genetics. 2019;211(2):495–502. doi:10.1534/genetics.118.301595. 30591514 PMC6366910

[jkad147-B10] Caruso AM , Serbina N, Klein E, Triebold K, Bloom BR, Flynn JL. Mice deficient in CD4 T cells have only transiently diminished levels of IFN-γ, yet succumb to tuberculosis. J Immunol. 1999;162(9):5407–5416. doi:10.4049/jimmunol.162.9.5407. 10228018

[jkad147-B11] Caws M , Thwaites G, Dunstan S, Hawn TR, Lan NTN, Thuong NTT, Stepniewska K, Huyen MNT, Bang ND, Tran HL, et al The influence of host and bacterial genotype on the development of disseminated disease with *Mycobacterium tuberculosis*. PLoS Pathog. 2008;4(3):e1000034. doi:10.1371/journal.ppat.1000034. 18369480 PMC2268004

[jkad147-B12] Chackerian AA , Behar SM. Susceptibility to *Mycobacterium tuberculosis*: lessons from inbred strains of mice. Tuberculosis(Edinb). 2003;83(5):279–285. doi:10.1016/S1472-9792(03)00017-9. 12972341

[jkad147-B13] Chesler EJ , Lu L, Shou S, Qu Y, Gu J, Wang J, Hsu HC, Mountz JD, Baldwin NE, Langston MA, et al Complex trait analysis of gene expression uncovers polygenic and pleiotropic networks that modulate nervous system function. Nat Genet. 2005;37(3):233–242. doi:10.1038/ng1518. 15711545

[jkad147-B14] Chesler EJ , Lu L, Wang J, Williams RW, Manly KF. WebQTL: rapid exploratory analysis of gene expression and genetic networks for brain and behavior. Nat Neurosci. 2004;7(5):485–486. doi:10.1038/nn0504-485. 15114364

[jkad147-B15] Chick JM , Munger SC, Simecek P, Huttlin EL, Choi K, Gatti DM, Raghupathy N, Svenson KL, Churchill GA, Gygi SP. Defining the consequences of genetic variation on a proteome-wide scale. Nature. 2016;534(7608):500–505. doi:10.1038/nature18270. 27309819 PMC5292866

[jkad147-B16] Cooper AM , Dalton DK, Stewart TA, Griffin JP, Russell DG, Orme IM. Disseminated tuberculosis in interferon gamma gene-disrupted mice. J Exp Med. 1993;178(6):2243–2247. doi:10.1084/jem.178.6.2243. 8245795 PMC2191280

[jkad147-B17] Cooper AM , Magram J, Ferrante J, Orme IM. Interleukin 12 (IL-12) is crucial to the development of protective immunity in mice intravenously infected with *Mycobacterium tuberculosis*. J Exp Med. 1997;186(1):39–45. doi:10.1084/jem.186.1.39. 9206995 PMC2198958

[jkad147-B18] Daniel J , Maamar H, Deb C, Sirakova TD, Kolattukudy PE. *Mycobacterium tuberculosis* uses host triacylglycerol to accumulate lipid droplets and acquires a dormancy-like phenotype in lipid-loaded macrophages. PLoS Pathog. 2011;7(6):e1002093. doi:10.1371/journal.ppat.1002093. 21731490 PMC3121879

[jkad147-B19] DeJesus MA , Ambadipudi C, Baker R, Sassetti C, Ioerger TR. TRANSIT—a software tool for Himar1 TnSeq analysis. PLoS Comput Biol. 2015;11(10):e1004401. doi:10.1371/journal.pcbi.1004401. 26447887 PMC4598096

[jkad147-B20] Dibbern J , Eggers L, Schneider BE. Sex differences in the C57BL/6 model of Mycobacterium tuberculosis infection. Sci Rep. 2017;7(1):10957. doi:10.1038/s41598-017-11438-z. 28887521 PMC5591305

[jkad147-B21] Flynn JL , Chan J, Triebold KJ, Dalton DK, Stewart TA, Bloom BR. An essential role for interferon γ in resistance to *Mycobacterium tuberculosis* infection. J Exp Med. 1993;178(6):2249–2254. doi:10.1084/jem.178.6.2249. 7504064 PMC2191274

[jkad147-B22] Gel B , Serra E. Karyoploter: an R/Bioconductor package to plot customizable genomes displaying arbitrary data. Bioinformatics. 2017;33(19):3088–3090. doi:10.1093/bioinformatics/btx346. 28575171 PMC5870550

[jkad147-B23] Gopal R , Monin L, Slight S, Uche U, Blanchard E, Fallert Junecko BA, Ramos-Payan R, Stallings CL, Reinhart TA, Kolls JK, et al Unexpected role for IL-17 in protective immunity against hypervirulent *Mycobacterium tuberculosis* HN878 infection. PLoS Pathog. 2014;10(5):e1004099. doi:10.1371/journal.ppat.1004099. 24831696 PMC4022785

[jkad147-B24] Hamilton BA , Yu BD. Modifier genes and the plasticity of genetic networks in mice. PLoS Genet. 2012;8(4):e1002644. doi:10.1371/journal.pgen.1002644. 22511884 PMC3325199

[jkad147-B25] Hernández-Pando R , Marquina-Castillo B, Barrios-Payán J, Mata-Espinosa D. Use of mouse models to study the variability in virulence associated with specific genotypic lineages of *Mycobacterium tuberculosis*. Infect Genet Evol. 2012;12(4):725–731. doi:10.1016/j.meegid.2012.02.013. 22426109

[jkad147-B26] Hershkovitz I , Donoghue HD, Minnikin DE, Besra GS, Lee OY-C, Gernaey AM, Galili E, Eshed V, Greenblatt CL, Lemma E, et al Detection and molecular characterization of 9000-year-old *Mycobacterium tuberculosis* from a neolithic settlement in the Eastern Mediterranean. PLoS One. 2008;3(10):e3426. doi:10.1371/journal.pone.0003426. 18923677 PMC2565837

[jkad147-B27] Houben RMGJ , Dodd PJ. The global burden of latent tuberculosis infection: a Re-estimation using mathematical modelling. PLOS Med. 2016;13(10):e1002152. doi:10.1371/journal.pmed.1002152. 27780211 PMC5079585

[jkad147-B28] Jaisinghani N , Dawa S, Singh K, Nandy A, Menon D, Bhandari PD, Khare G, Tyagi A, Gandotra S. Necrosis driven triglyceride synthesis primes macrophages for inflammation during *Mycobacterium tuberculosis* infection. Front Immunol. 2018;9:1490. doi:10.3389/fimmu.2018.01490 3. 30018616 PMC6037689

[jkad147-B29] Jinich A , Zaveri A, DeJesus MA, Flores-Bautista E, Smith CM, Sassetti CM, Rock JM, Ehrt S, Schnappinger D, Ioerger TR, et al 2021. The *Mycobacterium tuberculosis* transposon sequencing database (MtbTnDB): a large-scale guide to genetic conditional essentiality. bioRxiv 2021.03.05.434127. 10.1101/2021.03.05.434127. PMC12242109 40527579

[jkad147-B30] Kapopoulou A , Lew JM, Cole ST. The MycoBrowser portal: a comprehensive and manually annotated resource for mycobacterial genomes. Tuberculosis. 2011;91(1):8–13. doi:10.1016/j.tube.2010.09.006. 20980200

[jkad147-B31] Keller C , Hoffmann R, Lang R, Brandau S, Hermann C, Ehlers S. Genetically determined susceptibility to tuberculosis in mice causally involves accelerated and enhanced recruitment of granulocytes. Infect Immun. 2006;74(7):4295–4309. doi:10.1128/IAI.00057-06. 16790804 PMC1489748

[jkad147-B32] Kramnik I , Dietrich WF, Demant P, Bloom BR. Genetic control of resistance to experimental infection with virulent *Mycobacterium tuberculosis*. Proc Natl Acad Sci. 2000;97(15):8560–8565. doi:10.1073/pnas.150227197. 10890913 PMC26987

[jkad147-B33] Lavebratt C , Apt AS, Nikonenko B V, Schalling M, Schurr E. Severity of tuberculosis in mice is linked to distal chromosome 3 and proximal chromosome 9. J Infect Dis. 1999;180(1):150–155. doi:10.1086/314843. 10353873

[jkad147-B34] Li J , Guan X, Shaw N, Chen W, Dong Y, Xu X, Li X, Rao Z. Homotypic dimerization of a maltose kinase for molecular scaffolding. Sci Rep. 2014;4(1):6418. doi:10.1038/srep06418. 25245657 PMC4171701

[jkad147-B35] Long JE , DeJesus M, Ward D, Baker RE, Ioerger T, Sassetti CM. Identifying essential genes in *Mycobacterium tuberculosis* by global phenotypic profiling. Methods in Molecular Biology. New York: Humana Press Inc; 2015. p. 79–95. 10.1007/978-1-4939-2398-4_6 25636614

[jkad147-B36] Marquis JF , Nantel A, LaCourse R, Ryan L, North RJ, Gros P. Fibrotic response as a distinguishing feature of resistance and susceptibility to pulmonary infection with *Mycobacterium tuberculosis* in mice. Infect Immun. 2008;76(1):78–88. doi:10.1128/IAI.00369-07. 17938213 PMC2223652

[jkad147-B37] Medina E , North RJ. Resistance ranking of some common inbred mouse strains to *Mycobacterium tuberculosis* and relationship to major histocompatibility complex haplotype and Nramp1 genotype. Immunology. 1998;93(2):270–274. doi:10.1046/j.1365-2567.1998.00419.x. 9616378 PMC1364188

[jkad147-B38] Mishra BB , Lovewell RR, Olive AJ, Zhang G, Wang W, Eugenin E, Smith CM, Phuah JY, Long JE, Dubuke ML, et al Nitric oxide prevents a pathogen-permissive granulocytic inflammation during tuberculosis. Nat Microbiol. 2017;2(7):17072. doi:10.1038/nmicrobiol.2017.72. 28504669 PMC5461879

[jkad147-B39] Mitsos L-M , Cardon LR, Fortin A, Ryan L, LaCourse R, North RJ, Gros P. Genetic control of susceptibility to infection with *Mycobacterium tuberculosis* in mice. Genes Immun. 2000;1(8):467–477. doi:10.1038/sj.gene.6363712. 11197687

[jkad147-B40] Mitsos L-M , Cardon LR, Ryan L, LaCourse R, North RJ, Gros P. Susceptibility to tuberculosis: a locus on mouse chromosome 19 (*Trl-4*) regulates *Mycobacterium tuberculosis* replication in the lungs. Proc Natl Acad Sci U S A. 2003;100(11):6610–6615.. doi:10.1073/pnas.1031727100. 12740444 PMC164495

[jkad147-B41] Moreira-Teixeira L , Redford PS, Stavropoulos E, Ghilardi N, Maynard CL, Weaver CT, Freitas do Rosário AP, Wu X, Langhorne J, O’Garra A. T cell–derived IL-10 impairs host resistance to *Mycobacterium tuberculosis* infection. J Immunol. 2017;199(2):613–623. doi:10.4049/jimmunol.1601340. 28584007 PMC5502318

[jkad147-B42] Moreira-Teixeira L , Tabone O, Graham CM, Singhania A, Stavropoulos E, Redford PS, Chakravarty P, Priestnall SL, Suarez-Bonnet A, Herbert E, et al Mouse transcriptome reveals potential signatures of protection and pathogenesis in human tuberculosis. Nat Immunol. 2020;21(4):464–476. doi:10.1038/s41590-020-0610-z. 32205882 PMC7116040

[jkad147-B43] Munger SC , Raghupathy N, Choi K, Simons AK, Gatti DM, Hinerfeld DA, Svenson KL, Keller MP, Attie AD, Hibbs MA, et al RNA-Seq alignment to individualized genomes improves transcript abundance estimates in multiparent populations. Genetics. 2014;198(1):59–73. doi:10.1534/genetics.114.165886. 25236449 PMC4174954

[jkad147-B44] Nambi S , Long JE, Mishra BB, Baker R, Murphy KC, Olive AJ, Nguyen HP, Shaffer SA, Sassetti CM. The oxidative stress network of *Mycobacterium tuberculosis* reveals coordination between radical detoxification systems. Cell Host Microbe. 2015;17(6):829–837. doi:10.1016/j.chom.2015.05.008. 26067605 PMC4465913

[jkad147-B45] Parker CC , Dickson PE, Philip VM, Thomas M, Chesler EJ. Systems genetic analysis in GeneNetwork.org. Curr Protoc Neurosci. 2017;79(1):8.39.1–8.39.20. doi:10.1002/cpns.23. PMC5548442 28398643

[jkad147-B46] Peirce JL , Lu L, Gu J, Silver LM, Williams RW. A new set of BXD recombinant inbred lines from advanced intercross populations in mice. BMC Genet. 2004;5(1):7. doi:10.1186/1471-2156-5-7. 15117419 PMC420238

[jkad147-B47] Rosenberg OS , Dovey C, Tempesta M, Robbins RA, Finer-Moore JS, Stroud RM, Cox JS. Espr, a key regulator of *Mycobacterium tuberculosis* virulence, adopts a unique dimeric structure among helix-turn-helix proteins. Proc Natl Acad Sci. 2011;108(33):13450–13455. doi:10.1073/pnas.1110242108. 21795602 PMC3158157

[jkad147-B48] Roy S , Schmeier S, Kaczkowski B, Arner E, Alam T, Ozturk M, Tamgue O, Parihar SP, Kawaji H, Itoh M, et al Transcriptional landscape of *Mycobacterium tuberculosis* infection in macrophages. Sci Rep. 2018;8(1):6758. doi:10.1038/s41598-018-24509-6. 29712924 PMC5928056

[jkad147-B49] Sasani TA , Ashbrook DG, Beichman AC, Lu L, Palmer AA, Williams RW, Pritchard JK, Harris K. A natural mutator allele shapes mutation spectrum variation in mice. Nature. 2022;605(7910):497–502. doi:10.1038/s41586-022-04701-5. 35545679 PMC9272728

[jkad147-B50] Sassetti CM , Boyd DH, Rubin EJ. Genes required for mycobacterial growth defined by high density mutagenesis. Mol Microbiol. 2003;48(1):77–84. doi:10.1046/j.1365-2958.2003.03425.x. 12657046

[jkad147-B51] Sassetti CM , Rubin EJ. Genetic requirements for mycobacterial survival during infection. Proc Natl Acad Sci U S A. 2003;100(22):12989–12994. doi:10.1073/pnas.2134250100. 14569030 PMC240732

[jkad147-B52] Saul MC , Philip VM, Reinholdt LG, Center for Systems Neurogenetics of Addiction, Chesler EJ. High-diversity mouse populations for complex traits. Trends Genet. 2019;35(7):501–514. doi:10.1016/j.tig.2019.04.003. 31133439 PMC6571031

[jkad147-B53] Skelly DA , Czechanski A, Byers C, Aydin S, Spruce C, Olivier C, Choi K, Gatti DM, Raghupathy N, Keele GR, et al Mapping the effects of genetic variation on chromatin state and gene expression reveals loci that control ground state pluripotency. Cell Stem Cell. 2020;27(3):459–469.e8. doi:10.1016/j.stem.2020.07.005. 32795400 PMC7484384

[jkad147-B54] Sklar JG , Makinoshima H, Schneider JS, Glickman MS. *M. tuberculosis* intramembrane protease Rip1 controls transcription through three anti-sigma factor substrates. Mol Microbiol. 2010;77(3):605–617. doi:10.1111/j.1365-2958.2010.07232.x. 20545848 PMC3008510

[jkad147-B55] Smith CM , Baker RE, Proulx MK, Mishra BB, Long JE, Park SW, Lee H-N, Kiritsy MC, Bellerose MM, Olive AJ, et al Host–pathogen genetic interactions underlie tuberculosis susceptibility in genetically diverse mice. eLife. 2022;11:e74419. doi:10.7554/eLife.74419. 35112666 PMC8846590

[jkad147-B56] Smith CM , Sassetti CM. Modeling diversity: do homogeneous laboratory strains limit discovery? Trends Microbiol. 2018;26(11):892–895. doi:10.1016/j.tim.2018.08.002. 30166218 PMC6610874

[jkad147-B57] Taylor B , Heiniger H, Meier H. Genetic analysis of resistance to cadmium-induced testicular damage in mice. Proc Soc Exp Biol Med. 1973;143(3):629–633. doi:10.3181/00379727-143-37380. 4719448

[jkad147-B58] Taylor BA , Wnek C, Kotlus BS, Roemer N, MacTaggart T, Phillips SJ. Genotyping new BXD recombinant inbred mouse strains and comparison of BXD and consensus maps. Mamm Genome. 1999;10(4):335–348. doi:10.1007/s003359900998. 10087289

[jkad147-B59] Tsuyuguchi K , Suzuki K, Matsumoto H, Tanaka E, Amitani R, Kuze F. Effect of oestrogen on *Mycobacterium avium* complex pulmonary infection in mice. Clin Exp Immunol. 2001;123(3):428–434. doi:10.1046/j.1365-2249.2001.01474.x. 11298130 PMC1906003

[jkad147-B60] Wang R , Kreutzfeldt K, Botella H, Vaubourgeix J, Schnappinger D, Ehrt S. Persistent *Mycobacterium tuberculosis* infection in mice requires PerM for successful cell division. eLife. 2019;8:e49570. doi:10.7554/eLife.49570. 31751212 PMC6872210

[jkad147-B61] Wang X , Pandey AK, Mulligan MK, Williams EG, Mozhui K, Li Z, Jovaisaite V, Quarles LD, Xiao Z, Huang J, et al Joint mouse–human phenome-wide association to test gene function and disease risk. Nat Commun. 2016;7(1):10464. 26833085 10.1038/ncomms10464 PMC4740880

[jkad147-B62] Wheeler FC , Fernandez L, Carlson KM, Wolf MJ, Rockman HA, Marchuk DA. QTL Mapping in a mouse model of cardiomyopathy reveals an ancestral modifier allele affecting heart function and survival. Mamm Genome. 2005;16(6):414–423. doi:10.1007/s00335-005-2468-7. 16075368

[jkad147-B63] Wheeler FC , Tang H, Marks OA, Hadnott TN, Chu P-L, Mao L, Rockman HA, Marchuk DA. *Tnni3k* modifies disease progression in murine models of cardiomyopathy. PLoS Genet. 2009;5(9):e1000647. doi:10.1371/journal.pgen.1000647. 19763165 PMC2731170

[jkad147-B64] Whitaker M , Ruecker N, Hartman T, Klevorn T, Andres J, Kim J, Rhee K, Ehrt S. Two interacting ATPases protect *Mycobacterium tuberculosis* from glycerol and nitric oxide toxicity. J Bacteriol. 2020;202(16):e00202-20. doi:10.1128/JB.00202-20. 32482725 PMC8404711

[jkad147-B65] WHO . Global Tuberculosis Report 2022. Geneva. 2022.

[jkad147-B66] Wilburn KM , Meade RK, Heckenberg EM, Dockterman J, Coers J, Sassetti CM, Olive AJ, Smith CM. Differential requirement for IRGM proteins during tuberculosis infection in mice. Infect Immun. 2023;91(2):e0051022. doi:10.1128/iai.00510-22. 36629440 PMC9933630

[jkad147-B67] Wiltshire SA , Leiva-Torres GA, Vidal SM. Quantitative trait locus analysis, pathway analysis, and consomic mapping show genetic variants of Tnni3k, Fpgt, or H28 control susceptibility to viral myocarditis. J Immunol. 2011;186(11):6398–6405. doi:10.4049/jimmunol.1100159. 21525387

[jkad147-B68] Wu Y , Williams EG, Dubuis S, Mottis A, Jovaisaite V, Houten SM, Argmann CA, Faridi P, Wolski W, Kutalik Z, et al Multilayered genetic and omics dissection of mitochondrial activity in a mouse reference population. Cell. 2014;158(6):1415–1430. doi:10.1016/j.cell.2014.07.039. 25215496 PMC4179868

[jkad147-B69] Yan B-S , Kirby A, Shebzukhov YV, Daly MJ, Kramnik I. Genetic architecture of tuberculosis resistance in a mouse model of infection. Genes Immun. 2006;7(3):201–210. doi:10.1038/sj.gene.6364288. 16452998

[jkad147-B70] Zak DE , Penn-Nicholson A, Scriba TJ, Thompson E, Suliman S, Amon LM, Mahomed H, Erasmus M, Whatney W, Hussey GD, et al A blood RNA signature for tuberculosis disease risk: a prospective cohort study. Lancet. 2016;387(10035):2312–2322. doi:10.1016/S0140-6736(15)01316-1. 27017310 PMC5392204

[jkad147-B71] Zhang J , Lan Y, Li MY, Lamers MM, Fusade-Boyer M, Klemm E, Thiele C, Ashour J, Sanyal S. Flaviviruses exploit the lipid droplet protein AUP1 to trigger lipophagy and drive virus production. Cell Host Microbe. 2018;23(6):819–831.e5. doi:10.1016/j.chom.2018.05.005. 29902443

