# Supplementary material for: Genome-wide screen identifies host loci that modulate Mycobacterium tuberculosis fitness in immunodivergent mice
Source: G3 (Bethesda). 2023 Jul 5;13(9):jkad147. doi: 10.1093/g3journal/jkad147 (PMC10468300; doi:10.1093/g3journal/jkad147)
Supplement: jkad147_Supplementary_Data [file jkad147_supplementary_data.zip › Figure_S1_G3-2023-404171.pdf]

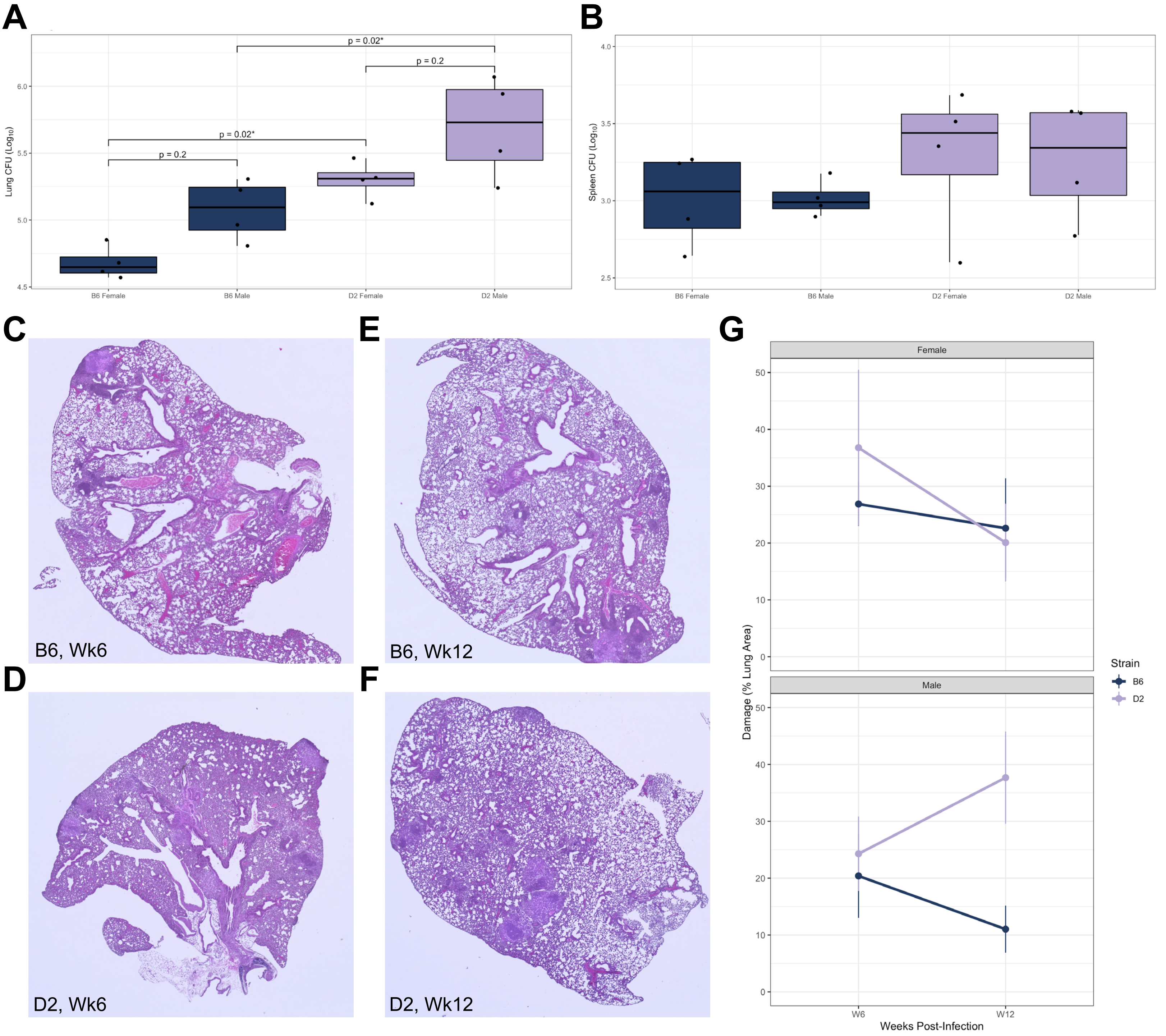

Figure S1: Sex has a significant effect on susceptibility by aerosol but is not dependent upon host genotype. (**A**) Both sex (p = 0.007) and genotype (p = 0.0003) have a significant effect on lung burden at 6 weeks post-infection by ANOVA. p-values included in the plot were calculated by Tukey's *posthoc* test. (**B**) Neither sex nor genotype significantly impacted spleen burden at 6 weeks post-infection by ANOVA. (**C**-**F**) Female B6 and D2 H&E-stained lung sections taken 6- and 12-weeks post-infection, 2X magnification, representative of n = 4 per genotype per timepoint. (**G**) Damage quantification of H&E-stained lung sections in QuPath v0.3.2 using an artificial neural network-based damage identification algorithm (n = 4 per strain, sex, and timepoint).
