# Supplementary material for: Genome-wide screen identifies host loci that modulate Mycobacterium tuberculosis fitness in immunodivergent mice
Source: G3 (Bethesda). 2023 Jul 5;13(9):jkad147. doi: 10.1093/g3journal/jkad147 (PMC10468300; doi:10.1093/g3journal/jkad147)
Supplement: jkad147_Supplementary_Data [file jkad147_supplementary_data.zip › Figure_S2_G3-2023-404171.pdf]

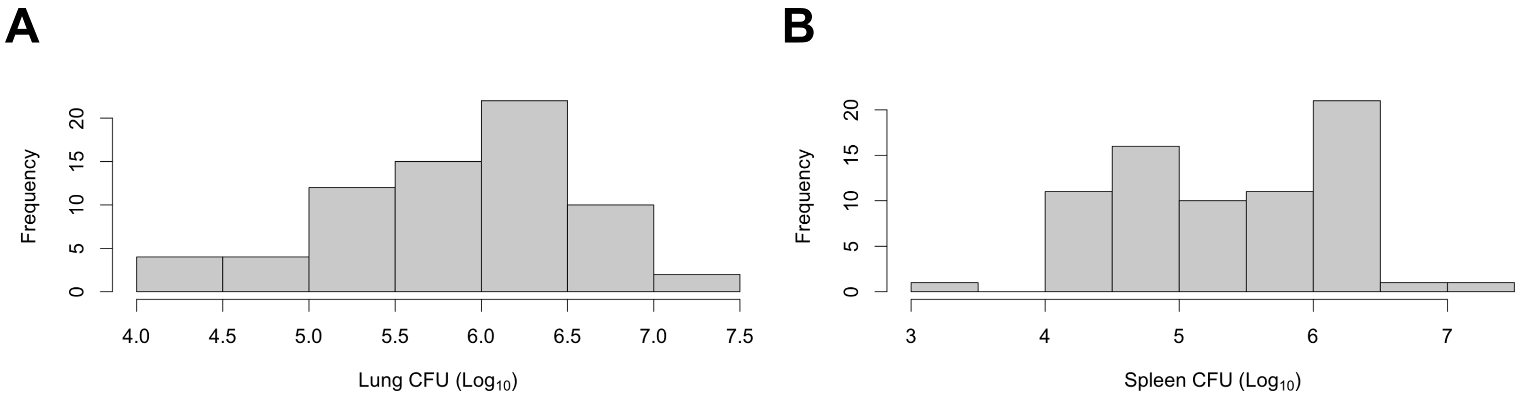


**Figure S2: Distribution of lung and spleen burden across the BXD panel at 4 weeks post-infection.** Histograms of lung (**A**) and spleen (**B**) burden values quantified across each screened BXD individual.
