# Supplementary material for: Genome-wide screen identifies host loci that modulate Mycobacterium tuberculosis fitness in immunodivergent mice
Source: G3 (Bethesda). 2023 Jul 5;13(9):jkad147. doi: 10.1093/g3journal/jkad147 (PMC10468300; doi:10.1093/g3journal/jkad147)
Supplement: jkad147_Supplementary_Data [file jkad147_supplementary_data.zip › Figure_S3_G3-2023-404171.pdf]

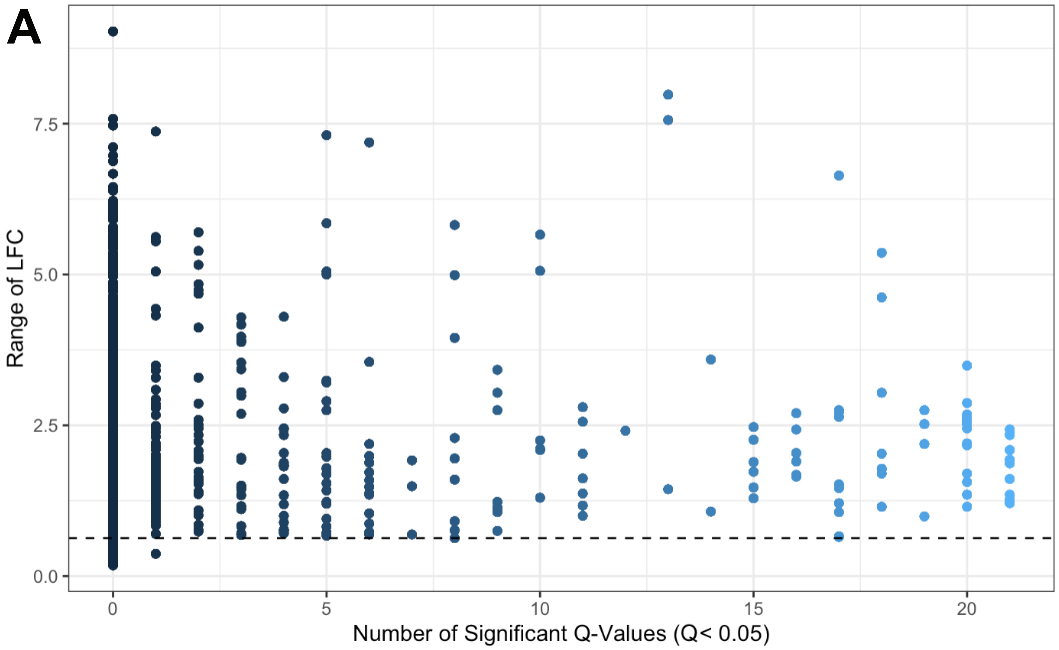

Figure S3: Thresholding criteria of transposon mutant fitness profiles for QTL mapping. To conduct QTL mapping on sufficiently varying bacterial genes, we excluded transposon mutants with a dynamic range of less than or equal to 0.63 $\log_2$ fold change (LFC), which is the minimum range of *Mtb* mutants with at least one significant Q-value between *in vitro* and *in vivo* conditions across the panel.
