# Supplementary material for: Genome-wide screen identifies host loci that modulate Mycobacterium tuberculosis fitness in immunodivergent mice
Source: G3 (Bethesda). 2023 Jul 5;13(9):jkad147. doi: 10.1093/g3journal/jkad147 (PMC10468300; doi:10.1093/g3journal/jkad147)
Supplement: jkad147_Supplementary_Data [file jkad147_supplementary_data.zip › Figure_S4_G3-2023-404171.pdf]

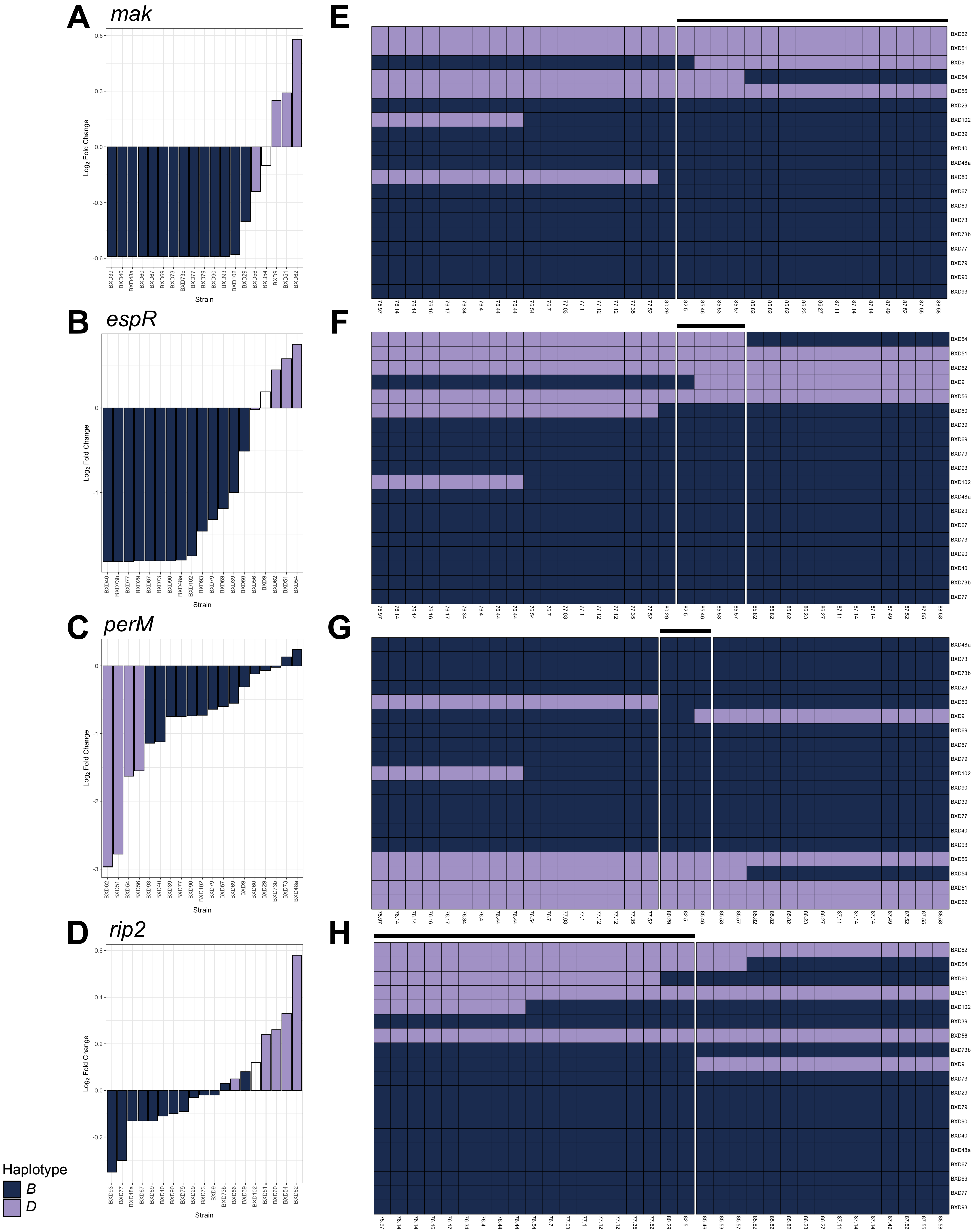

Figure S4: BXD haplotypes within the chromosome 6 QTL hotspot. Bar plots comparing the predicted haplotypes of each BXD strain at the QTL position for (**A**) *mak*, (**B**) *espR*, (**C**) *perM*, and (**D**) *rip2* with the fitness of each transposon mutant within each BXD genotype. Empty bars represent haplotype states that could not be assessed with at least 95% confidence at the QTL. Visualizations of the BXD predicted haplotypes across the hotspot interval sorted top to bottom from highest to lowest mutant fitness of (**E**) *mak*, (**F**) *espR*, (**G**) *perM*, and (**H**) *rip2* transposon mutants. Position values are represented in Mb. The black bar above each plot denotes the location of the 95% Bayesian confidence interval of each QTL within the hotspot.
